# Untargeted metabolomics unveil alterations of biomembranes permeability in human HaCaT keratinocytes upon 60 GHz millimeter-wave exposure

**DOI:** 10.1038/s41598-019-45662-6

**Published:** 2019-06-27

**Authors:** Pierre Le Pogam, Yann Le Page, Denis Habauzit, Mickael Doué, Maxim Zhadobov, Ronan Sauleau, Yves Le Dréan, David Rondeau

**Affiliations:** 1 Univ Rennes, CNRS, IETR (Institut d’Électronique et de Télécommunication de Rennes), UMR 6164, F-35000 Rennes, France; 2 Univ Rennes, Inserm, EHESP, Irset (Institut de recherche en santé, environnement et travail) – UMR_S 1085, F-35000 Rennes, France; 3 Département de Chimie, Université de Bretagne Occidentale, 6 avenue Victor Le Gorgeu, 29238 Brest, Cedex France

**Keywords:** Metabolomics, Diagnostic markers

## Abstract

A joint metabolomic and lipidomic workflow is used to account for a potential effect of millimeter waves (MMW) around 60 GHz on biological tissues. For this purpose, HaCaT human keratinocytes were exposed at 60.4 GHz with an incident power density of 20 mW/cm², this value corresponding to the upper local exposure limit for general public in the context of a wide scale deployment of MMW technologies and devices. After a 24h-exposure, endo- and extracellular extracts were recovered to be submitted to an integrative UPLC-Q-Exactive metabolomic and lipidomic workflow. R-XCMS data processing and subsequent statistical treatment led to emphasize a limited number of altered features in lipidomic sequences and in intracellular metabolomic analyses, whatever the ionization mode (*i.e* 0 to 6 dysregulated features). Conversely, important dysregulations could be reported in extracellular metabolomic profiles with 111 and 99 frames being altered upon MMW exposure in positive and negative polarities, respectively. This unexpected extent of modifications can hardly stem from the mild changes that could be reported throughout transcriptomics studies, leading us to hypothesize that MMW might alter the permeability of cell membranes, as reported elsewhere.

## Introduction

Owing to the saturation of the lower part of the microwave spectrum and to the growing demand for higher data rates, the millimeter waves (MMW) are increasingly used for wireless communications, *i.e*. for Wireless Local/Personal/Body Area Networks (WLAN, PAN, BAN) or in the context of the upcoming 5 G mobile standard^1^. These electromagnetic radiations correspond to frequencies ranging from 30 to 300 GHz (free-space wavelengths spanning from 10 to 1 mm). Interestingly, MMW have been used for therapeutic purposes indicating that physiological processes might be altered upon exposure to these radiations^2^. Accordingly, the assessment of the potential effects of MMW on human health is thus of paramount importance prior to the wide scale deployment of technologies exploiting this band. MMW are known for their shallow penetration of human tissues (between a few tenth of millimeter to a millimeter), making skin keratinocytes and free nerve endings the primary targets for MMW^3^. It is assumed that some general effects of MMW may be initiated by the release of skin-secreted factors into the blood stream or through the stimulation of superficial free nerve endings^4,5^.

The shallow penetration depth of MMW in skin, results in elevated levels of Specific Absorption Rates (SAR) compared to lower microwave frequencies with the same Incident Power Density (IPD). As a consequence, this leads to a noticeable local heating for IPD exceeding typically 5 mW/cm^2^ ^6^. Accordingly, one of the main safety concerns regarding these frequencies is the local heating of skin caused by the absorption of MMW energy in the human body^7^. Whether electromagnetic-field specific effects occur upon exposure to MMW^8–12^ or not^13–17^ has been a matter of debate for a long period of time. Besides the warm-up effect, the mechanisms accounting for non-thermal effects occurring at low-power exposure have not been fully demonstrated yet so that their understanding remains an open challenge^18^. Consequently, the guidelines established by the International Commission on Non Ionizing Radiation Protection (ICNIRP) aim at circumventing thermal hazards associated with MMW exposure. This led to define two exposure scenarios. For far-field exposure, the IPD is limited to 1 mW/cm². For body-centric wireless networks that are related to very limited exposition area, the IPD limit is set at 20 mW/cm² (averaged over 1 cm²). While such exposure scenarios may trigger a heat-shock response, it is assumed that the thermoregulation due to blood flow effect might be efficient to avoid any thermal damage.

Notwithstanding decades of investigations and a significant number of studies dedicated at unravelling the effects of MMW on biosystems, the number of reliable and reproducible experimental data remains scarce, most likely stemming for difficulties in dissociating thermal and electromagnetically pure effects. Our group aimed at evaluating the biological effects of MMW in the 60 GHz band. Previous *in vitro* studies revealed that MMW at 57–64 GHz exert no influence on protein homeostasis for IPD low enough to prevent any temperature increase^19–21^. Moreover, transcriptomic studies highlighted no, or very weak, effect of MMW on keratinocyte gene expression under athermal conditions^22–24^. By contrast, it must be mentioned that some authors have pointed out the MMW effects on cell membranes, indicating that permeability changes could be induced by a direct effect on membrane proteins or phospholipids domain organization^25^. The possibility of permeation across the lipid bilayer led us to consider the metabolomic profiling of human keratinocytes as a pertinent next step to better understand the interactions between MMW radiation and cell membrane in the context of human body exposure to 60 GHz waves. The goal of the present study is to apply an untargeted metabolomic strategy based on UHPLC-HRMS assessment of metabolic changes appearing upon exposure to MMW in human HaCaT keratinocytes cell lines. Cells were exposed at 60.4 GHz with an IPD of 20 mW/cm² with the temperature being artificially maintained constant between non-exposed and exposed samples^26^. As metabolites represent the sharp end of systems biology, including multiple up-stream steps, the keratinocytes were exposed for 24 hours to enable possible changes. Then, to afford as wide a coverage of HaCaT keratinocytes chemistry as possible, lipidomic and metabolomic profilings were performed in both intra- and extracellular fractions in positive and negative polarities using a metabolomic workflow that we had previously validated^27^.

## Results and Discussion

### Data quality assurance

The analytical strategy adopted herein was based on two different chromatographic separation methods (HILIC and RPLC, see experimental section) coupled to detection in mass spectrometry involving both positive and negative-ion modes. This strategy was applied by distinguishing the endo- and exocellular fractions as well to deepen the coverage of the metabolome. As a result, this led to analyze each biological sample 8 times, in distinct sessions of analyses.

Prior to any data processing or analysis, several quality checks were adopted (*i.e* column pressure checking, variations of internal standards in QC/study samples and instrumental stability in terms of retention time and accurate masses) along the whole batch. Accordingly, internal standards were spiked into each sample prior to LC-HRMS analyses to assess retention time stability, the consistency of signal intensities and mass accuracy along the whole batch. No significant drift in retention time could be evidenced. Likewise, standard accurate mass measurements errors always remained below 5 ppm. For metabolomic analyses, the selected external standards were leucine-d_3_ (RT 5.97 min), tryptophan-d_3_ (RT 6.10 min), indole acetic acid-d_5_ (RT, 1.81 min) and tetradecanedioic acid-d_24_ (RT 1.38 min). Creatine-d_3_ (RT 8.22 min) and L-lysine-d_4_ (RT 14.31 min) were selected as internal standards. As to lipidomic sequences, phosphatidylcholine (15:0) (RT 8.37 min), lysophosphatidylcholine (15:0) (RT 1.75 min), ceramide (d18:1) (RT 17.95 min), C15:0 (RT 3.19 min) and C23:0 (RT 9.27 min) were used as external standards, whereas C17:0 (RT 4.30 min) served as an internal standard. All these standards revealed a coefficient of variation below 30% except in the case of C17:0 which displayed a slightly higher value in negative-ion mode of the exo-lipidomics sequence (33.7%) (Tables S1 and S2).

The analysis of QC samples served as a further criterion to establish both the quality of the generated data and the stability of the instrumental platform. For this purpose, it is highly recommended that QC peak tables should pass pre-determined criteria, a widely admitted one being that a majority of features (*i.e*. 70%) shows a coefficient of variation less than 30%^28^. With respect to this parameter, most sequences displayed satisfying values, with only negative-ion mode exo-lipidomics sequence exhibiting a slightly lower value of 56.3% (Table 1). The stability of the analytical instrumentation could also be assessed based on the clustering of the QC samples in a Principal Component Analysis (PCA) score plot of all tested sequences.

**Table 1:** Validation of the generated data set using R-XCMS data processing.

Metabolome	Fraction	Ion mode	Ions	Ions with CV QC < 30% (% of total ions)	Ions with both CV QC < 30% and FC > 2
Exo	Lipido	Positive	3081	2161 (70.82)	289
		Negative	1920	1126 (56.25)	126
	Metabo	Positive	2750	2076 (75.42)	662
		Negative	1632	1263 (77.37)	506
Endo	Lipido	Positive	4295	3920 (91.27)	540
		Negative	2468	2164 (87.68)	264
	Metabo	Positive	1910	1525 (79.84)	210
		Negative	1017	843 (83.00)	58

Altogether, these parameters underscore both the robustness of the LC-HRMS system operating conditions and the validity of the retrieved metabolomic data, paving the way for their statistical processing.

### Lipidomic RPLC-MS analyses

**Figure 1:**
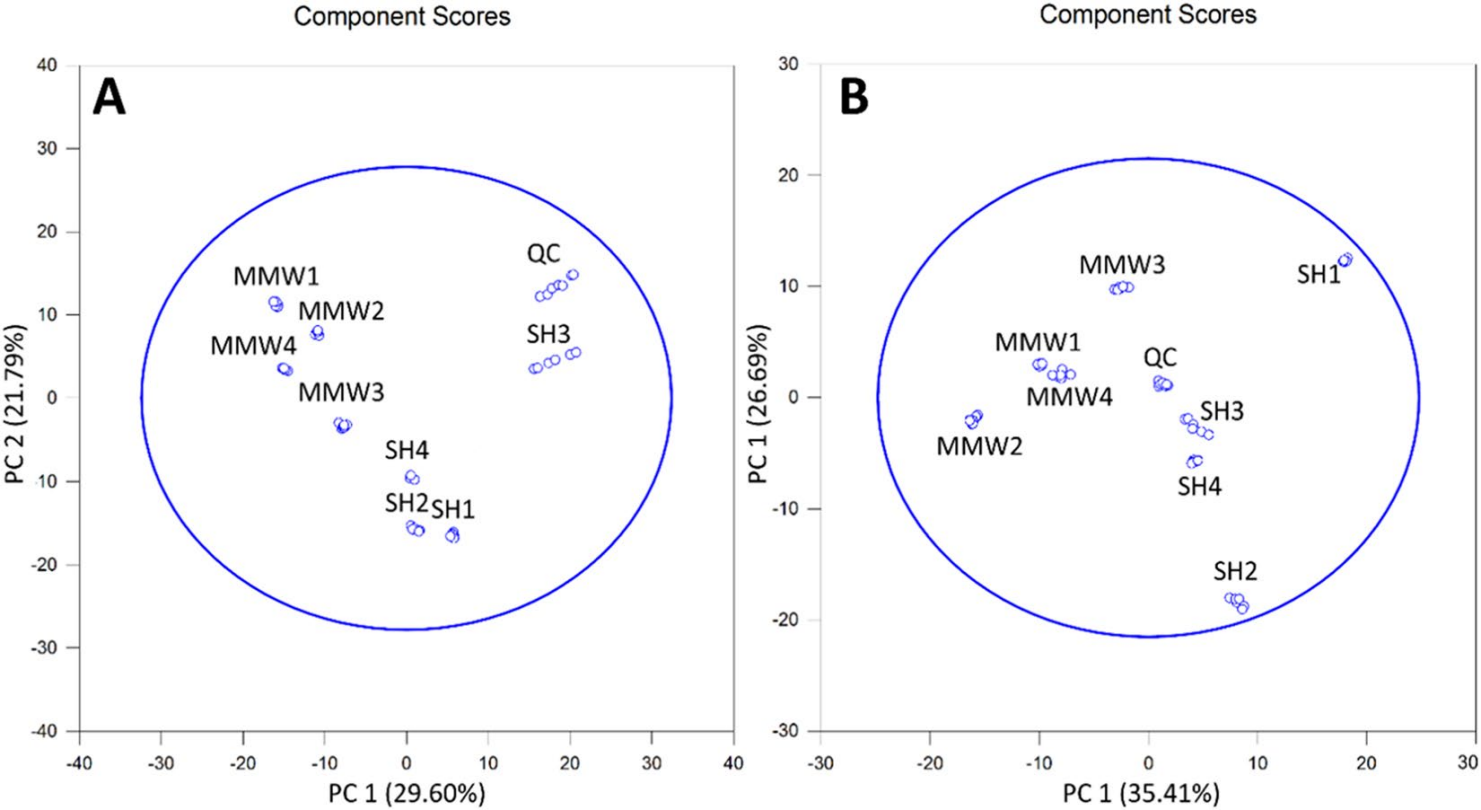
PCA plots generated from endocellular lipidomic sequences from the features displaying both a Fold-Change ≥2 and a p-value < 0.05 upon R-XCMS data processing performed from UHPLC-HRMS sequences recorded in (**A**) Positive-Ion Mode and (**B**) Negative-Ion Mode. Note that SH_*i*_ (with *i* = 1 to 4) and MMW_*j*_ (with *j* = 1 to 4) are related to the non-exposed and exposed samples, respectively; QC represents the overall of the quality control samples.

As to intracellular fractions, the filtered data subsets were represented by 540 and 264 features, for positive and negative ionization polarities respectively. Non-exposed and exposed samples clustered separately, being discriminated along PC1 component in these two sequences (Fig. 1).

In positive and negative polarities, univariate analyses respectively identified six and four masses as the main discriminators between the two sample groups (Figs S1 and S2). Regarding positive-ion mode, tentative hits could be retrieved for two out of five species, which might correspond to ceramide derivatives and/or N-palmitoylsphingosine derivatives (Table S3). Putative matches could be proposed for two ions appearing as dysregulated in negative-ion mode, tentatively corresponding to a further ceramide derivative (Cer (d18:0/16:0)) and to a diglyceride derivative DG (36:3) (Table S4). Besides the very limited number of features appearing as dysregulated, one should note that their corresponding Fold-Change indices display modest values remaining below 5 for all features identified in the course of negative-ion mode and below 10 for those pinpointed throughout positive-ion mode analyses. The only exception was M453T283 in this latter group, for which no putative identification could be retrieved.

**Figure 2:**
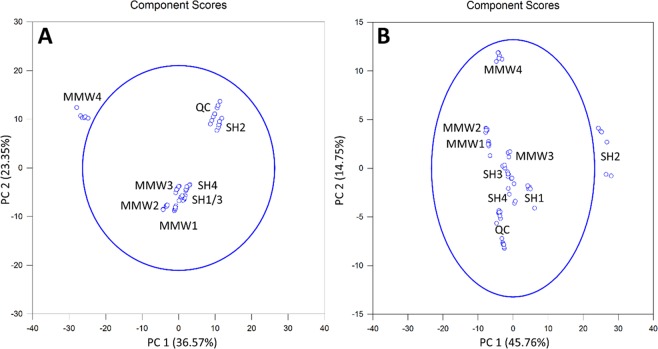
PCA plots yielded by exocellular lipidomic analyses from the ions having a Fold-Change ≥2 with a p-value < 0.05 upon R-XCMS data processing performed from UHPLC-HRMS sequences recorded in (**A**) Positive-Ion Mode and (**B**) Negative-Ion Mode. Note that SH_*i*_ (with *i* = 1 to 4) and MMW_*j*_ (with *j* = 1 to 4) are related to the non-exposed and exposed samples, respectively; QC represents the overall of the quality control samples.

Filtered RPLC datasets related to extracellular fractions consisted of 289 and 126 features in positive and negative-ion modes, respectively. In both ionization modes, PCA plots accounted for roughly 60% of the total variance between the two groups. However, whatever the considered ionization mode, these plots did not lead to a clear-cut discrimination of the samples according to their exposure status (Fig. 2).

While no discriminating feature could be highlighted during the course of negative-ion mode data processing, two ions of interest could however be evidenced in positive polarity, *i.e*. M627T51 and M783T478 (Fig. S3). Putative hits could only be retrieved for this latter feature that displayed an important FC of 764, which corresponds to a phosphatidylcholine derivative with a sum composition of (36:4) (Table S5).

### Metabolomic HILIC analyses

**Figure 3:**
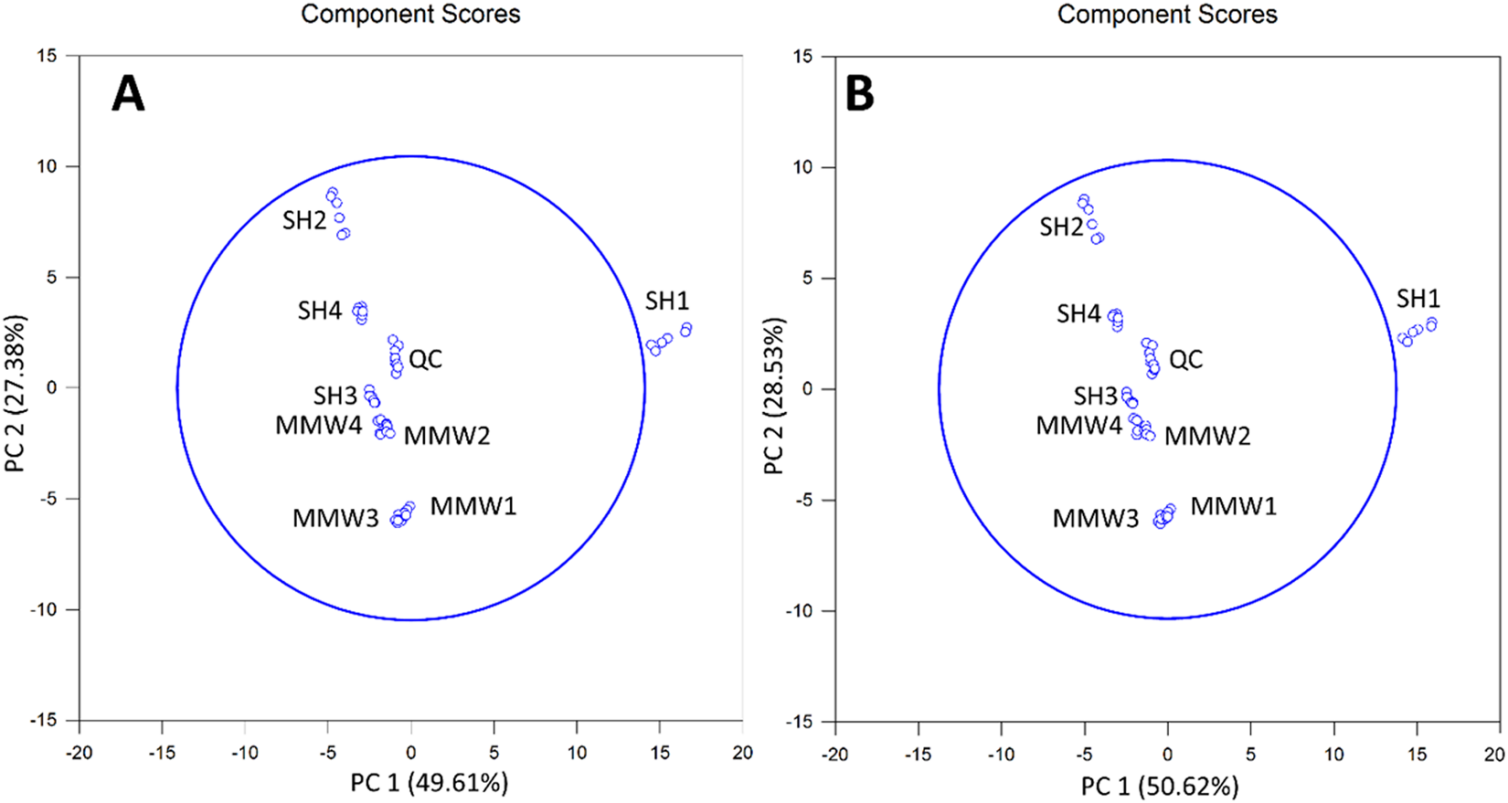
PCA plots obtained from endocellular metabolomic analyses from the features displaying both a Fold-Change ≥2 and a p-value < 0.05 upon R-XCMS data processing performed from UHPLC-HRMS sequences recorded in (**A**) Positive-Ion Mode and (**B**) Negative-Ion Mode. Note that SH_*i*_ (with *i* = 1 to 4) and MMW_*j*_ (with *j* = 1 to 4) are related to the non-exposed and exposed samples, respectively; QC represents the overall of the quality control samples.

Endocellular data subset with features satisfying both FC > 2 and ANOVA p-value < 0.05 were respectively represented by 210 and 58 features in positive and negative-ion modes. Generated PCA plots roughly represented 75% of the total variance and could discriminate between non-exposed and exposed samples along PC2 component in both ion modes (Fig. 3).

Notwithstanding this discrimination, the statistical processing workflow did not lead to emphasize any relevant biomarker in the negative-ion mode data set. Positive-ion mode endometabolomics only resulted in highlighting one biomarker with a moderate FC value of 2.8 for which two putative identifications could be retrieved from HMDB database (*i.e*. either 2-aminoheptanedioic acid or N-carboxyethyl-γ-aminobutyric acid) (Fig. S4 and Table S6).

**Figure 4:**
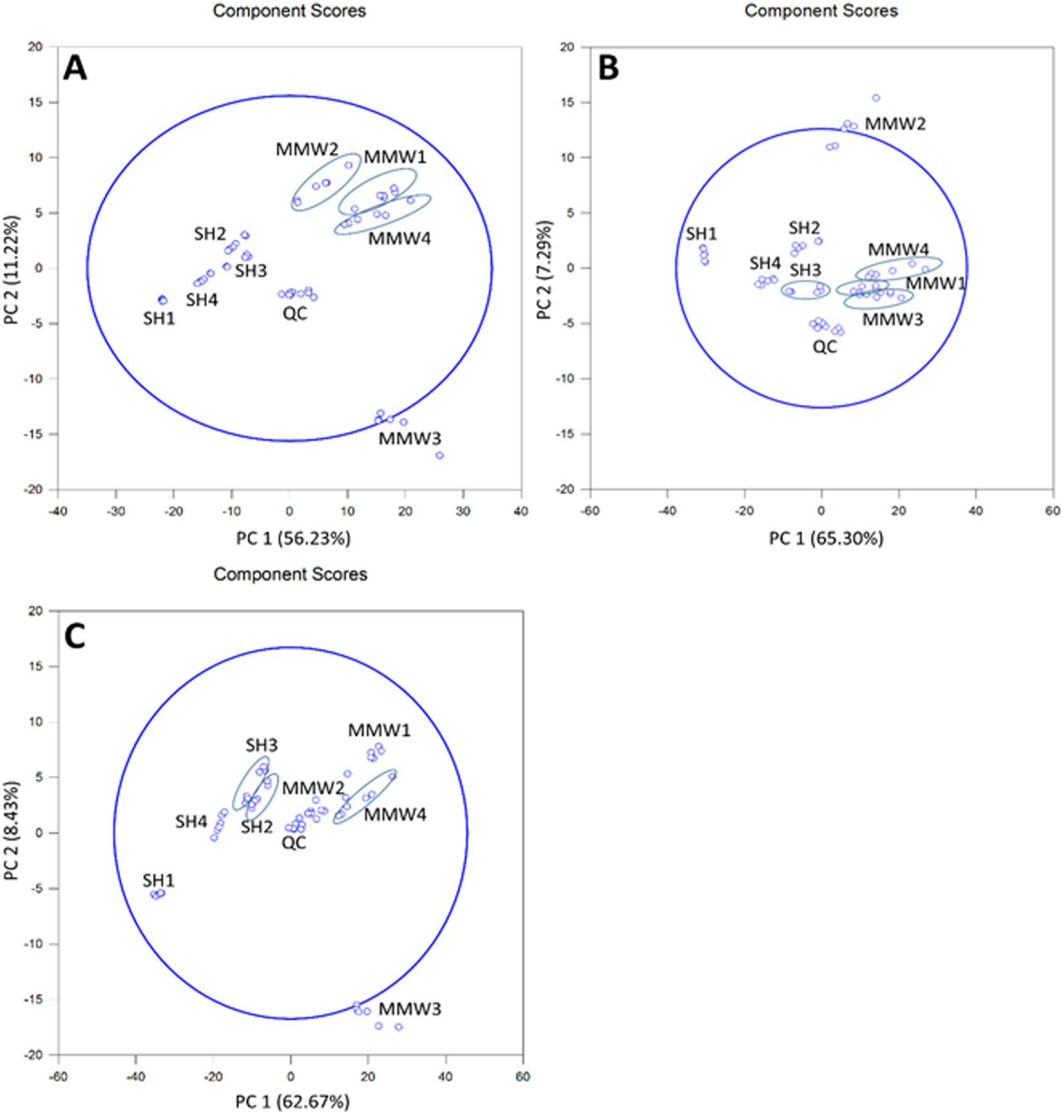
PCA plots of exocellular metabolomics sequences, selecting ions having both a FC > 2 and a p-value < 0.05 upon R-XCMS data processing performed from UHPLC-HRMS sequences recorded in (**A**,**B**) Positive-Ion Mode and (**C**) Negative-Ion Mode. Note that SH_*i*_ (with *i* = 1 to 4) and MMW_*j*_ (with *j* = 1 to 4) are related to the non-exposed and exposed samples, respectively; QC represents the overall of the quality control samples.

PCA plots related to exometabolomic sequences revealed clear-cut discriminations according to millimeter wave exposure status in both polarities. Owing to the large number of features in the filtered data subset in positive-ion mode (662), the frames were split into two halves prior to generating the PCA plots after being ordered by increasing masses. For both these data subsets, PCA plots roughly accounted for 70% of the total variance (*ca*. 60% for PC1 and ~ 9% for PC2). In these two groups, the samples could be discriminated in a straightforward manner along PC1 component (Fig. 4A,B). Regarding negative-ion mode, the PCA plot (engulfing 506 features) accounted for 74.1% of the total variance among the two groups, where PC1 and PC2 had respective contributions of 62.7 and 8.4%. Control and exposed samples also exhibited an obvious separation along PC1 dimension (Fig. 4C).

The careful validation of both extracted ion chromatograms and box-and-whisker plot as described in the Data Processing section led applied to retainment of 111 and 99 features as dysregulated in positive and negative polarities, respectively (Figs S5 and S6). The current study was performed using a single HRMS analyser, so that the identification of the dysregulated features could only rely on exact mass data, including any limitation coming along with it in terms of structural resolution. Resultantly, the structural assignments remain tentative and most often do not lead to reach unambiguous metabolites. From a practical viewpoint, handling an array of references covering the structural diversity of candidate biomarkers is not a realistic purpose in the frame of a metabolomic study pinpointing such an elevated number of features of interest. Despite this structural uncertainty, these putative identifications reveal that dysregulated features encompass an array of structurally diverse metabolites (Tables S7 and S8). Emphasizing such a large extent of dysregulations, especially in the exo-metabolomic sequences, appears as an intriguing outcome. Indeed, as upstream transcriptomics analyses did not highlight significant alterations in gene expression^23^, such dramatic changes would not have been expected. This led us to assume that these modifications might not be related to modifications of enzyme expression but rather stem from alterations in membrane permeability. Cell membranes are regarded as major targets for the interactions between millimeter waves and biological systems since a variety of bioeffects were reported upon exposure to these radiofrequencies^25^. As an example, 60 GHz exposure with an incident power density of 0.9 mW/cm² (*i.e*. in the typical range of values expected from wireless communications) was proved to induce structural modifications of artificial biomembranes. Consequently, MMW exposure was demonstrated to increase the lateral pressure of phospholipid monolayers although not strongly enough to disturb phospholipid microdomain organization in biomembranes^29^. Further biochemical processes could be evidenced such as the externalization of phosphatidylserine, even though the biological relevance of this event remains to be determined^30^. Likewise, 53-GHz radiations and 130-GHz pulse modulated exposures were shown to alter the permeability of phospholipid vesicles^31–33^. MMW action on the orientation of charged and dipolar molecules in the region located between the aqueous phase and the hydrocarbon interior of the membrane has been hypothesized to represent the driving force to rearrange phospholipids bilayers, resulting in an increase of small molecules permeability across the membrane^33^. This new organization of the bilayer presumably displays a higher curvature which would elicit metabolites leakage. It can also be assumed that electromagnetic radiations of specific frequencies might excite components of cell membrane, depending on their electric dipoles and possibly leading to form Bose-condensed phonons^34^. Such assumptions are consistent with structural changes observed in cells upon MMW exposure, with different deformations being reported in bacteria^35^.

Likewise, it can be assumed that a similar phenomenon might trigger the leakage of intracellular metabolites into the extracellular medium shown in this manuscript. This inference is further strengthened by the huge majority of dysregulated features which are found to be upregulated in the treated group (107/111 and 98/99 in positive and negative-ion modes, respectively. It is interesting to note that under different exposure conditions, membrane permeabilization upon electric pulses of nanosecond duration were previously reported to occur in mammalian cells^36,37^.

## Conclusion

As far as can be ascertained, this report represents the first metabolomic investigation focusing on the effects of MMW. To get as wide an insight into cellular processes as possible, a joint metabolomic and lipidomic profiling strategy was designed and the extra and intracellular contents were discriminated. It appeared that all lipidomic sequences and intracellular metabolomic profiles were slightly affected by MMW but drastic changes in extracellular metabolomic sequences could be evidenced. During these experiments, we put great emphasis on controlling cell culture parameters (temperature, pH, incubator humidity). Much attention has been paid to temperature control, however, it cannot be ruled out that unexpected changes at subcellular scale have occurred and could be responsible for the differences found. Moreover, by making the choice to put the non-exposed control in the same place in our exposure system, we ensure that these cells are in the same growing conditions that those that are exposed. Nevertheless, it should be noted that this strategy has the disadvantage of inducing a shift of one day of culture between the control and the exposed samples. While the unusually high number of dysregulated features makes of their unambiguous structural assignment an unrealistic purpose, the current study enables drawing significant and unprecedented conclusions as to the effects of MMW exposure on cellular systems, especially when combining them with former studies carried out by our group. Accordingly, as upstream pan-transcriptomic studies in this cellular system did not led to emphasize any significant change upon 60-GHz MMW exposure, it is reasonable to assume that the vast amount of dysregulations reported in these sequences do not stem from alterations of gene expression but rather from alterations in membrane permeability, consistently with previous reports on acellular phospholipidic systems. The tentative metabolites identified throughout the current workflow might serve as a ground to focus on subsets of metabolites through the so-called target-based metabolomics. For this purpose, multiplex LC-MS-MRM pipelines proved useful for the quantitative profiling of some of the hundreds of expected metabolites in complex biological samples with no structural/identity ambiguity. Based on the current findings, such follow-up studies could be limited to the exocellular compartment. Finally, we can conclude that our model, purely *in vitro*, haven’t to be lead to a direct extrapolation of our results at the organism level. In the future, further studies will be necessary to assess MMW bioeffects on animal models and to investigate potential dysregulations induced by lower IPD values prior to the wide-scale deployment of technologies based on these specific frequencies.

## Methods

### Chemicals, reagents and materials

LC-MS grade water, methanol (MeOH), methyl tert-butyl ether (MTBE), chloroform (CHCl_3_), acetonitrile (MeCN), 2-propanol (IPA), ammonium acetate and acetic acid were purchased from Sigma-Aldrich (St. Louis, MO, USA). The standard mixtures Calmix-positive (*i.e*. caffeine, L-methionyl-arginyl-phenylalanylalanine acetate and Ultramark 1621) and Calmix-negative (*i.e*. acetic acid, sodium dodecyl sulfate, taurocholic acid sodium salt hydrate and Ultramark 1621), were purchased from Thermo Fisher Scientific (Waltham, MA, USA).

1,2-Dipentadecanoyl-sn-glycero-3-phosphocholine (PC(15:0/15:0)), 1-pentadecanoyl-2-hydroxy-sn-glycero-3-phosphocholine (Lyso PC(15:0)), 1,2-diheptadecanoyl-sn-glycero-3-phosphoethanolamine (PE(17:0/17:0)) and N-heptadecanoyl-D-erythro-sphingosine (Cer(d18:1/17:0)) were obtained from Coger (Paris, France). _L_-Tryptophan-2,3,3-d_3_ (Trypto-d_3_), indole-2,4,5,6,7-d_5_-3-acetic acid (Ind-AA-d_5_), 1,14-tetradecanedioic-d_24_ acid (Tetra-A-d_24_) were purchased from Cluzeau Info Labo (Sainte-Foy-La-Grande, France). 1,2,3-triheptadecanoyl-glycerol (TG (17:0)), pentadecanoic acid (C15:0), tricosanoic acid (C23:0), heptadecanoic acid (C17:0), leucine-5,5,5-d_3_ (Leu-d_3_), creatine-(methyl-d_3_) (Crea-d_3_) and lysine-4,4,5,5-d_4_ (Lys-d_4_) were purchased from Sigma-Aldrich (St. Louis, MO, USA).

Stock standard solutions (1 mg/L) were prepared in CHCl_3_ (for lipidic compounds) or in MeOH and kept at −20 °C. The internal standard (IS) solution contains C17:0, Crea-d_3_ and Lys-d_4_ at 10 ng/µL in a MeOH/H_2_O mixture (4/1). Lipidomic external standard (ES) solution containing (PC (15:0/15:0), Lyso PC (15:0), PE (17:0/17:0), TG (17:0), Cer (d18:1/17:0), C15:0 and C23:0 at 0.5 ng/µL and metabolomic (ES) solution containing Leu-d_3_, Trypto-d_3_, Ind-AA-d_5_ and Tetra-A-d_24_ at 1 ng/µL were subsequently prepared in CHCl_3_ (lipidomics ES) or in MeOH (metabolomics ES).

### Cell culture

A human keratinocyte cell line (HaCaT) was cultured as described elsewhere^22^. In an attempt to circumvent any senescence or drift of the cellular populations, keratinocytes were exposed at early passages (between 10 and 16). To enable proper cross-sample comparison, the quantity of cellular material was estimated through the evaluation of total ERK protein amount (Western Blot using anti ERK1 (K-23) antibody (Santa Cruz Biotechnology, Dallas, Texas, USA))^38^. The exposure medium designed to keep pH buffering in the non-gassed incubator of the exposure system as described elsewhere^39^. Briefly, it consists in powder reconstituted DMEM medium, completed with fetal calf serum, 4-(2-hydroxyethyl)-1- piperazineethanesulfonic acid (HEPES) and antibiotics.

### Experimental setup for sample exposition

Sample irradiation was performed as previously reported^24^. A thorough description of the exposure system can be found in Zhadobov *et al*.^19^. Briefly, a 6-well culture plate was positioned in a MEMMERT UE400 incubator to be exposed from the bottom by a standard pyramidal horn antenna. Cells were then irradiated or not by this antenna with an average incident power density (IPD) of 20 mW/cm² for 24 hours, according to the guidelines established by the International Commission on Non-Ionizing Radiation Protection (ICNIRP) for MMW with limited exposition area^40^. Both MMW-exposures and non-exposed samples were performed inside the same incubator, on different days. To mitigate exposure variability between sample groups, the non-exposed and MMW-Exposed cell samples were located in the same position within the same incubator^39^. To avoid a thermal effect associated with MMW exposure, the temperature increase was counteracted by lowering the incubator set point by the predicted increment. Consistently with literature^23^, MMW exposure at such IPD resulted in an elevation of 8 °C leading to set the temperature of the incubator at 28 °C in the MMW group to reach 36 °C, as in non-exposed cells. Assuming that thermal convection currents occurring in the radiofrequency-exposed groups may lead to a differential condensation of the culture medium, the consistency of the pH value was monitored in both non-exposed and exposed sample groups. An identical pH value of 7.7 could be measured from the different culture media, irrespective of their exposure status. HaCaT keratinocytes cell viability was formerly reported to be of 100% for pH values spanning across the 7.0–8.2 range^41^.

### Metabolomics workflow

**Figure 5:**
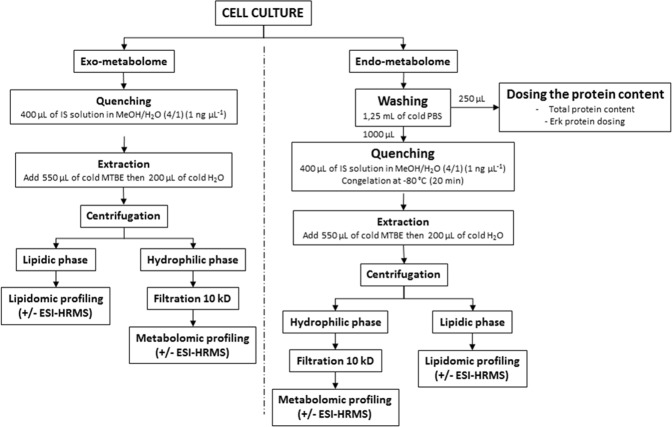
Schematic workflow of the sample preparation procedure^27^.

As MMW exposure might result in evidencing little to no biological effect, it was very important to validate the ability of the retained metabolomic workflow to emphasize the dysregulations triggered by a known interfering agent. This preliminary study, carried out using the cytotoxic drug 2-deoxyglucose, demonstrated the adequacy of the proposed workflow for metabolomics purposes^27^. The whole sample preparation workflow is summarized in Fig. 5.

Metabolomic analyses were carried out on 4 independent biological samples for both non-exposed and MMW-exposed groups. As to extracellular profiling, a 100-µL aliquot of the culture medium was recovered for further sample processing. Regarding intracellular metabolite assessment, the cells were washed with 1 mL of phosphate buffer solution after having discarded the remaining culture medium. The cells were subsequently detached by scraping within 1250 µL of PBS solution. A 250 µL-aliquot was recovered to evaluate the amount of total ERK, a ubiquitous protein, by western blotting. Both the endo- and exo-cellular fractions were spiked with 400 µL of Internal Standard solution dissolved in MeOH/H_2_O (4/1, *v/v*). Endocellular fractions were frozen at −80 °C for 20 minutes to facilitate cell membrane disruption. Solutions were then added with 550 µL of MTBE and vortexed three times for ten seconds (separated by one minute breaks in ice), 200 µL of cold water were then added with the same vortexing sequence being repeated. After centrifugation of the solutions (12,000 g, 4 °C, 15 min), a 300 µL-aliquot of the upper organic phase was transferred to a vial to be spiked with 40 µL of the lipidomics ES solution (0.5 ng/µL) in CHCl_3_ prior to being dried under N_2_ flux. The dry extract was dissolved in 200 µL of MeCN/IPA/H_2_O solution (65:30:5, *v/v/v*). Likewise, a 300 µL-aliquot of the lower aqueous phase was recovered and centrifugally filtered through a Millipore 10 kDa cutoff filter (12,000 g, 4 °C, 20 min). The solution was evaporated to dryness under N_2_ and later resuspended in 200 µL of a MeCN/H_2_O (9/1, *v/*v) solution.

QC samples were obtained by pooling 20 µL aliquots from the test samples to represent a bulk control sample. The samples were stored at −80 °C pending UHPLC-HRMS analysis.

### UHPLC/HRMS analyses

Analyses were performed using an ultrahigh performance liquid chromatography (Waters Acquity), hyphenated with a Thermo QExactive mass spectrometer. Samples were injected (5 µL) onto either an Acquity CSH C_18_ column (1.7 µm, 2.1 × 100 mm; Waters) for lipidomic sequences or a SeQuant ZIC-HILIC column (3.5 µm, 2.1 × 100 mm; Merck) for metabolomic HILIC analyses. The standard mobile phases for RPLC (lipidomic sequences) were A = MeCN/H_2_O/ammonium acetate 1 M/acetic acid (600/390/10/1, *v*/*v*/*v*/*v*) and B = IPA/MeCN/H_2_O/ammonium acetate 1 M/acetic acid (880/100/10/10/1, *v*/*v*/*v*/*v*/*v*). For HILIC conditions, the mobile phases were A = H_2_O/ammonium acetate 1 M/acetic acid (980/10/1, *v*/*v*/*v*) and B = MeCN/solvent A (950/50, *v*/*v*). The column oven temperature was kept constant at 35 °C for metabolomic HILIC runs and at 45 °C for lipidomic RPLC analyses. RPLC analyses were performed by gradient elution as follows: T, 0 min, 40% B; 0–2 min, 50% B linear; 2–12 min, 70% B linear; 12–17 min, 99% B linear; 17–25 min, 99% B; 25–29 min, 40% B. HILIC acquisitions were obtained using the following gradient program: T, 0–2 min, 95% B; 2–5 min, 80% B; 5–12 min, 60% B linear; 12–14 min, 40% B linear; 14–16 min, 40% B; 16–26 min, 95% B.

ESI source conditions were set as follows: sheath gas flow, 55 Arbitrary Units (AU); auxiliary gas flow, 10 AU; capillary temperature, 300 °C; spray voltage, either 3.5 kV (lipidomics) or 3.0 kV (metabolomics); S-lens radiofrequency, 50 AU. Mass spectra were either acquired over the *m/z* range 150–1500 (lipidomics) or 65–975 (metabolomics) at a resolving power of 35000 Full Width Half Maximum (FWHM) measured at *m/z* 200. The Automatic Gain Control (AGC target) was set at high dynamic range (5 × 10^5^) with a maximum injection time of 100 ms. External calibrations of the MS instrument were performed using the Calmix-positive and Calmix-negative standard solution for the positive and the negative ionization modes, respectively. Exact mass measurements did not take into account the mass of the electron.

The analytical run was initiated by a number of injections of QC samples to ensure that LC and MS systems had time to equilibrate and perform satisfactorily. Irrespective of their exposed or non-exposed sample status, the study samples were randomized to limit the effect of time trends and thus minimize bias introduced by non-biological parameters (*e.g*. instrumental drifts). Each sample was analyzed six times.

### Statistical processing

The statistical processing of the metabolomic data considered all exposed and unexposed samples independently of one another. LC/MS data were further processed by R package XCMS (version 3.2). The preprocessing results generated a data matrix as a feature list table comprising their integrated intensities (reconstructed ion chromatogram peak areas), along with the observed fold-changes and associated p-values^42^. Applied peak picking parameters were prefilter = c(5,25), snthresh = 6, mzdiff = 0.01 and ppm = 15. Initial alignment (bw = 20, minfrac = 0.66, minsamp = 4, mzvid = 0.008) and retention time correction (standard loess, plottype = c(deviation) were then applied. Further alignment steps were performed using the same processing parameters with decreasing bw values (lowered to bw = 9 for the second round and to 5 for the final stage). Subsequently, R-package CAMERA was used for peak annotation after XCMS data processing^43^. Consistently with reported guidelines, features found in less than 20% of the analyzed samples were removed according to the so-called 80% rule^44^.

As to univariate analyses, the coefficient of variation (CV) within QC samples was calculated by dividing the standard deviation by the mean intensity of each feature, leading to a histogram of the resulting CV distribution. Subsequently, computation of the Fold-Change (FC, ratio of abundance between non-exposed and exposed samples) along with the corresponding p-value (statistical significance from a Student t-test) streamlined the selection of metabolites of interest. Features selection was based on the following criteria: CV QC < 30%, FC > 2 and ANOVA p-value < 0.05. Extracted Ion Chromatograms (EICs) were individually monitored to exclude potential artifacts from the ion list. Later on, Box and Whiskers Plot related to all metabolites of potential interest were also individually checked to retain the features for which no overlap existed between values obtained from non-exposed and exposed samples. Such features were tentatively identified against databases as described later on. The top and bottom of each box represent the 25^th^ and 75^th^ percentiles, the center line indicates the median and the extent of the whiskers depicts the 5th and 95th percentiles. Regarding multivariate analyses, Principal Component Analyses (PCA) were performed to get a general overview of the interrelationship between study samples as well as QC. Statistical graphs were prepared using SigmaPlot 13.0® (Systat Software, Inc., USA). Filtered data sets related to each sequence (only retaining features displaying FC > 2 and p-value < 0.05) were then analyzed by PCA to explore samples’ relationship and grouping. For this purpose, PCA retrieves a small number of principal components that summarize the measured data to visualize trends and emphasize possible outliers.

### Metabolite identification

[M + H]^+^, [M-H_2_O + H]^+^, [M + Na]^+^ and [M-H_2_O + Na]^+^ were selected as possible adducts for positive polarity. Regarding negative-ion mode, [M-H]^−^, [M-H_2_O-H]^−^ and [M-H_2_O + HCOOH-H]^−^ were considered. Putative identifications were carried out against the freely available database Human Metabolome Data Base (HMDB). Whenever possible, efforts were made to narrow down identification possibilities among isobaric compounds according to elution order, matrix of occurrence.

## Supplementary information

Supporting Material

## Data Availability

The datasets generated and analysed during the current study are available from the corresponding author on reasonable request. The accession number for the metabolomics data reported in this paper are Massive MSV000083829.
