# Supplementary material for: Untargeted metabolomics unveil alterations of biomembranes permeability in human HaCaT keratinocytes upon 60 GHz millimeter-wave exposure
Source: Sci Rep. 2019 Jun 27;9:9343. doi: 10.1038/s41598-019-45662-6 (PMC6597695; doi:10.1038/s41598-019-45662-6)
Supplement: Supplementary file 1 — Supporting Material [file 41598_2019_45662_MOESM1_ESM.docx]

**Untargeted metabolomics unveil alterations of biomembranes permeability in human HaCaT keratinocytes upon 60 GHz millimeter-wave exposure**

Pierre Le Pogam^1^, Yann Le Page^2^, Denis Habauzit^2^, Mickael Doué^1^, Maxim Zhadobov^1^, Ronan Sauleau^1^, Yves Le Dréan^2^ and David Rondeau^1,3*^

^1^ Univ Rennes, CNRS, IETR (Institut d’Électronique et de Télécommunication de Rennes), UMR 6164, F-35000 Rennes, France.

^2^ Univ Rennes, Inserm, EHESP, Irset (Institut de recherche en santé, environnement et travail) – UMR_S 1085, F-35000 Rennes, France.

^3^ Département de Chimie, Université de Bretagne Occidentale, 6 avenue Victor Le Gorgeu, 29238 Brest, Cedex, France.

*Corresponding author:

David Rondeau, Institut d’Électronique et de Télécommunications de Rennes (IETR), UMR CNRS 6164, Université de Rennes 1, Campus de Beaulieu, 263 avenue du Général Leclerc, 35042 Rennes Cedex, France.

tel: +33 223235445, email: david.rondeau@univ-rennes1.fr

**Table S1** CV values of internal and external standards among QC samples in lipidomics sequences. *ND: Not Detected

| Lipidomics | Endo | | Exo | |
| --- | --- | --- | --- | --- |
| Ion mode | Positive | Negative | Positive | Negative |
| C15:0 | ND | 3.89 | ND | 10.40 |
| C17:0 | ND | 2.84 | ND | 33.71 |
| C23:0 | ND | 4.06 | ND | 16.37 |
| LysoPC(15:0) | 5.55 | 5.85 | 7.06 | 7.08 |
| Cer(d18:1) | 5.15 | 5.15 | 3.85 | 6.76 |
| PC(15:0) | 2.87 | 5.51 | 3.56 | 11.22 |
| PE(17:0) | 5.64 | 3.16 | 3.91 | 6.27 |
| TG(17:0) | 0.36 | ND | 4.15 | ND |

**Table S2** CV values of internal and external standards among QC samples in metabolomics sequences.

*ND Not Detected

| Metabolomics | Endo | | Exo | |
| --- | --- | --- | --- | --- |
| Ion mode | Positive | Negative | Positive | Negative |
| Crea-d3 | 3.30 | 4.20 | 12.70 | 5.72 |
| Leu-d3 | 1.29 | 2.09 | 5.74 | 5.18 |
| IndAA | ND | 3.07 | ND | 17.12 |
| Trypto-d3 | 2.31 | 2.48 | 5.30 | 5.23 |
| TetraAA-d24 | ND | 8.52 | ND | 26.04 |

**Table S3** Tentative identification of dysregulated metabolites under MMW exposure in positive-ion mode endo lipidomics sequence.

| Feature of interest | **HMDB hits (±0.003 Da)** | HMDB ID | LogP | FC | p-value |
| --- | --- | --- | --- | --- | --- |
| M326T127  Monoisotopic mass : 326.378223 | **No hit** | -- | -- | 8.3 | 2.6E-6 |
| M453T283  Monoisotopic mass : 452.519453 | **No hit** | -- | -- | 64.8 | 1.0E-6 |
| M521T615 [M-H_2_O+H]^+^  Monoisotopic mass : 537.516865  Calcd. mass : 537.512091 | **N-Palmitoylsphingosine** | HMDB00790 | 9.49/10.87 | 4.6 | 5.9E-10 |
|  | **Ceramide (d18:1/16:0)** | HMDB04949 | 9.49/10.87 |  |  |
| M561T615[M+Na]^+^  Monoisotopic mass : 537.512027  Calcd. mass : 537.512091 | **N-Palmitoylsphingosine** | HMDB00790 | 9.49/10.87 | 3.1 | 9.3E-15 |
|  | **Ceramide (d18:1/16:0)** | HMDB04949 | 9.49/10.87 |  |  |
| M597T615 [M+H]^+^  Monoisotopic mass : 595.565158  Calcd. mass : 595.590345 | **Ceramide (d18:0/20:0)** | HMDB11760 | 9.94/12.85 | 4.9 | 5.9E-11 |
| M925T1024  Monoisotopic mass : 924.802000 | **No hit** | -- | -- | 3.5 | 7.5E-15 |

**Table S4** Tentative identification of dysregulated metabolites under MMW exposure in negative-ion mode endocellular lipidomics.

| Feature of interest | **HMDB hits (±0.003 Da)** | HMDB ID | LogP | FC | p-value |
| --- | --- | --- | --- | --- | --- |
| M539T651  [M-H]^-^  Monoisotopic mass : 539.528303  Calcd. Mass : 539.527745 | **Ceramide (d18:0/16:0)** | HMDB11760 | 9.94/12.85 | 3.0 | 1.1E-14 |
| M599T615-2  [M-H_2_O-H]^-^  Monoisotopic mass : 618.522801  Calcd. Mass / 618.522325 | DG(14:1(9Z)/22:2(13Z,16Z)/0:0) | HMDB07059 | 10.06/12.7 | 2.8 | 8.9E-10 |
|  | DG(16:0/20:3(5Z,8Z,11Z)/0:0) | HMDB07110 | 10.17/12.7 |  |  |
|  | DG(16:0/20:3(8Z,11Z,14Z)/0:0) | HMDB07111 | 10.1/12.7 |  |  |
|  | DG(16:1(9Z)/20:2(11Z,14Z)/0:0) | HMDB07138 | 10.05/12.7 |  |  |
|  | DG(18:0/18:3(6Z,9Z,12Z)/0:0) | HMDB07162 | 10.14/12.7 |  |  |
|  | DG(18:0/18:3(9Z,12Z,15Z)/0:0) | HMDB07163 | 10.12/12.7 |  |  |
|  | DG(18:1(11Z)/18:2(9Z,12Z)/0:0) | HMDB07190 | 10.06/12.7 |  |  |
|  | DG(18:1(9Z)/18:2(9Z,12Z)/0:0) | HMDB07219 | 10.06/12.7 |  |  |
|  | DG(18:2(9Z,12Z)/18:1(11Z)/0:0) | HMDB07246 | 10.06/12.7 |  |  |
|  | DG(18:2(9Z,12Z)/18:1(9Z)/0:0) | HMDB07247 | 10.06/12.7 |  |  |
|  | DG(18:3(6Z,9Z,12Z)/18:0/0:0) | HMDB07274 | 10.14/12.7 |  |  |
|  | DG(18:3(9Z,12Z,15Z)/18:0/0:0) | HMDB07303 | 10.11/12.7 |  |  |
|  | DG(20:2(11Z,14Z)/16:1(9Z)/0:0) | HMDB07418 | 10.05/12.7 |  |  |
|  | DG(20:3(5Z,8Z,11Z)/16:0/0:0) | HMDB07446 | 10.17/12.7 |  |  |
|  | DG(20:3(8Z,11Z,14Z)/16:0/0:0) | HMDB07475 | 10.1/12.7 |  |  |
|  | DG(22:2(13Z,16Z)/14:1(9Z)/0:0) | HMDB07647 | 10.06/12.7 |  |  |
|  | DG(16:0/0:0/20:3n9) | HMDB56019 | 10.19/12.7 |  |  |
|  | DG(16:0/0:0/20:3n6) | HMDB56025 | 10.13/12.7 |  |  |
|  | DG(18:0/0:0/18:3n6) | HMDB56049 | 10.17/12.7 |  |  |
|  | DG(18:0/0:0/18:3n3) | HMDB56056 | 10.14/12.7 |  |  |
|  | DG(14:1n5/0:0/22:2n6) | HMDB56147 | 10.12/12.7 |  |  |
| M601T716  Monoisotopic mass : 600.513372 | **No hit** | -- | -- | 3.4 | 7.4E-12 |
| M625T716  Monoisotopic mass : 624.557648 | **No hit** | -- | -- | 2.7 | 5.2E-12 |

**Table S5** Tentative identification of dysregulated metabolites under MMW exposure in positive-ion mode exocellular lipidomics.

| Feature of interest | **HMDB hits (±0.003 Da)** | HMDB ID | LogP | FC | p-value |
| --- | --- | --- | --- | --- | --- |
| M627T51  Monoisotopic mass : 627.020929 | **No hit** | -- | -- | 5.2 | 2.2E-9 |
| M783T478  [M+H]^+^  Monoisotopic mass : 781.562761  Calcd. mass : 781.562155 | PC(14:0/22:4(7Z,10Z,13Z,16Z)) | HMDB07889 | 5.73/8.44 | 264 | 4.4E-11 |
|  | PC(16:0/20:4(5Z,8Z,11Z,14Z)) | HMDB07982 | 5.75/8.44 |  |  |
|  | PC(16:0/20:4(8Z,11Z,14Z,17Z)) | HMDB07983 | 5.72/8.44 |  |  |
|  | PC(16:1(9Z)/20:3(5Z,8Z,11Z)) | HMDB08013 | 5.73/8.44 |  |  |
|  | PC(16:1(9Z)/20:3(8Z,11Z,14Z)) | HMDB08014 | 5.68/8.44 |  |  |
|  | PC(18:0/18:4(6Z,9Z,12Z,15Z)) | HMDB08042 | 5.76/8.44 |  |  |
|  | PC(18:1(11Z)/18:3(6Z,9Z,12Z)) | HMDB08073 | 5.71/8.44 |  |  |
|  | PC(18:1(9Z)/18:3(6Z,9Z,12Z)) | HMDB08106 | 5.72/8.44 |  |  |
|  | PC(18:1(9Z)/18:3(9Z,12Z,15Z)) | HMDB08107 | 5.69/8.44 |  |  |
|  | PC(18:2(9Z,12Z)/18:2(9Z,12Z)) | HMDB08138 | 5.68/8.44 |  |  |
|  | PC(18:3(6Z,9Z,12Z)/18:1(11Z)) | HMDB08169 | 5.71/8.44 |  |  |
|  | PC(18:3(6Z,9Z,12Z)/18:1(9Z)) | HMDB08170 | 5.71/8.44 |  |  |
|  | PC(18:3(9Z,12Z,15Z)/18:1(11Z)) | HMDB08202 | 5.68/8.44 |  |  |
|  | PC(18:3(9Z,12Z,15Z)/18:1(9Z)) | HMDB08203 | 5.69/8.44 |  |  |
|  | PC(18:4(6Z,9Z,12Z,15Z)/18:0) | HMDB08234 | 5.75/8.44 |  |  |
|  | PC(20:3(5Z,8Z,11Z)/16:1(9Z)) | HMDB08365 | 5.73/8.44 |  |  |
|  | PC(20:3(8Z,11Z,14Z)/16:1(9Z)) | HMDB08398 | 5.75/8.44 |  |  |
|  | PC(20:4(5Z,8Z,11Z,14Z)/16:0) | HMDB08429 | 5.75/8.44 |  |  |
|  | PC(20:4(8Z,11Z,14Z,17Z)/16:0) | HMDB08462 | 5.73/8.44 |  |  |
|  | PC(22:4(7Z,10Z,13Z,16Z)/14:0) | HMDB08623 | 5.72/8.44 |  |  |

**Table S6** Tentative identification of dysregulated metabolites under MMW exposure in positive-ion mode endocellular metabolomics.

| Feature of interest | **HMDB hits (±0.003 Da)** | HMDB ID | LogP | FC | p-value |
| --- | --- | --- | --- | --- | --- |
| M176T497  [M+H]^+^  Monoisotopic mass : 175.084458  Calcd. Mass : 175.084458 | N-Carboxyethyl-g-aminobutyric acid | HMDB02201 | -2.5/-3.2 | 2.8 | 2.2E-6 |
|  | 2-Aminoheptanedioic acid | HMDB34252 | -3.2/-2.4 |  |  |

**Table S7** Tentative identification of dysregulated metabolites upon MMW exposure in positive-ion mode exocellular metabolomics.

| Feature of interest | **HMDB hits (±0.003 Da)** | HMDB ID | LogP | FC | p-value |
| --- | --- | --- | --- | --- | --- |
| M66T367  Monoisotopic mass : 66.256598 | **No hit** | -- | -- | 2.2 | 1.7E-9 |
| M69T423 [M-H_2_O+H]^+^  Monoisotopic mass : 86.077495  Calcd. mass : 86.073165 | **Iso-Valeraldehyde** | HMDB06478 | 1.29/1.05 | 2.4 | 5.3E-11 |
| M76T643 [M+H]^+^  Monoisotopic mass : 75.067985  Calcd. mass : 75.068414 | **Trimethylamine N-oxide** | HMDB00925 | -2.00/-0.93 | 2.2 | 2.4E-12 |
| M77T401  Monoisotopic mass : 77.038612 | **No hit** | -- | -- | 2.4 | 5.9E-11 |
| M86T451 [M-H_2_O+H]^+^  Monoisotopic mass : 103.103998  Calcd. mass : 103.099714 | **Neurine** | HMDB31259 | -1.00/-3.5 | 2.4 | 2.4E-8 |
| M102T230  Monoisotopic mass : 102.06653 | **No hit** | -- | -- | 2.1 | 4.7E-10 |
| M103T401  Monoisotopic mass : 103.054541 | **No hit** | -- | -- | 2.4 | 1.5E-11 |
| M105T986  Monoisotopic mass : 121.999981  Calcd. mass : 121.003765 | **3-Mercaptolactic acid** | HMDB02127 | -0.33/-0.42 | 2.9 | 10E-15 |
| M106T439 [M+H]^+^  Monoisotopic mass : 105.041108  Calcd. mass : 105.042593 | **L-Serine** | HMDB00187 | -3.4/-3.9 | 2.2 | 1.3E-10 |
|  | **D-Serine** | HMDB03406 | -3.4/-3.9 |  |  |
|  | **Protein Serine** | HMDB62263 | -3.4/-3.9 |  |  |
| M107T401 [M+Na]^+^  Monoisotopic mass : 84.0597225  Calcd. mass : 84.057515 | **Methyl propenyl ketone** | HMDB01184 | 1.48/1.25 | 2.5 | 1.3E-11 |
|  | **3-methyl-2-butenal** | HMDB12157 | 0.97/1.00 |  |  |
| M112T288 [M+H]^+^  Monoisotopic mass : 111.0429690  Calcd. mass : 111.043262 | **Cytosine** | HMDB00630 | -0.94/-1.10 | 3.4 | 1.9E-14 |
| M114T101  Monoisotopic mass : 114.037557 | **No hit** | -- | -- | 2.9 | 4.2E-14 |
| M114T313 [M+H]^+^  Monoisotopic mass : 113.058631  Calcd. mass : 113.058912 | **Creatinine** | HMDB00562 | -1.60/-1.10 | 2.6 | 6.6E-13 |
| M118T402 [M+H]^+^  Monoisotopic mass : 117.057543  Calcd. mass : 117.053826 | **Guanidoacetic acid** | HMDB00128 | -1.80/-3.10 | 2.4 | 4.5E-11 |
| M118T402 [M-H_2_O+H]^+^  Monoisotopic mass : 135.072823  Calcd. mass : 135.068414 | **2-Phenylacetamide** | HMDB10715 | 0.64/0.80 |  |  |
| M120T401/M120T451  Monoisotopic mass : 137.088484  /137.088519  Calcd. mass : 137.084064 | **Tyramine** | HMDB00306 | -0.14/0.68 | 2.4/2.7 | 1.8E-11/  0.0193 |
|  | **2-hydroxyphenylethylamine** | HMDB01065 | 0.31/0.47 |  |  |
| M122T816 [M-H_2_O+H]^+^  Monoisotopic mass : 139.078935  Calcd. mass : 139.074562 | **Histidinal** | HMDB12234 | -0.95/-1.20 | 2.6 | 3.7E-9 |
| M131T401 [M+H]^+^  Monoisotopic mass : 130.041613  Calcd. mass : 131.094629 | **L-Isoleucine** | HMDB00172 | -1.70/-1.50 | 2.5 | 8E-11 |
|  | **L-Alloisoleucine** | HMDB00557 | -1.70/-1.50 |  |  |
|  | **L-Leucine** | HMDB00687 | -1.80/-1.60 |  |  |
|  | **L-Norleucine** | HMDB01645 | -1.70/-1.40 |  |  |
|  | **N-methylvaline** | HMDB61716 | -1.80/-1.70 |  |  |
| M132T413/M132T451  Monoisotopic masses : 132.102290/132.102323 | **No hit** | -- | -- | 2.4/3.1 | 5.2E-11/  2.4E-12 |
| M136T313 [M+H]^+^  Monoisotopic mass : 135.040669  Calcd. mass : 135.035400 | **Homocysteine** | HMDB00742 | -2.30/-2.60 | 2.7 | 2.3E-12 |
|  | **Methylcysteine** | HMDB02108 | -2.20/-2.40 |  |  |
| M136T313 [M+Na]^+^  Monoisotopic mass : 113.058840  Calcd. mass : 113.058912 | **Creatinine** | HMDB00562 | -1.60/-1.10 |  |  |
| M136T313 [M-H_2_O+Na]^+^  Monoisotopic mass : 131.074120  Calcd. mass : 131.069477 | **Creatine** | HMDB00064 | -1.60/-2.90 |  |  |
| M137T214/M137T258  [M+H]^+^  Monoisotopic masses : 136.038327/136.038305  Calcd. mass : 136.037173 | **Erythronic acid** | HMDB00613 | -2.10/-2.10 | 3.0/  6.0 | 1.5E-13/  8.5E-7 |
|  | **Threonic acid** | HMDB00943 | -2.10/-2.10 |  |  |
|  | **L-Threonic acid** | HMDB62620 | -2.10/-2.10 |  |  |
| M137T214/M137T258  [M-H_2_O+Na]^+^  Monoisotopic masses : 132.071778/132.071756  Calcd. mass : 132.078644 | **2-hydroxy-3-methylpentanoic acid** | HMDB00317 | 0.74/0.86 |  |  |
|  | **(5R)-5-Hydroxyhexanoic acid** | HMDB00409 | 0.32/0.35 |  |  |
|  | **5-hydroxyhexanoic acid** | HMDB00525 | 0.32/0.35 |  |  |
|  | **D-Leucic acid** | HMDB00624 | 0.50/0.78 |  |  |
|  | **Leucinic acid** | HMDB00665 | 0.50/0.78 |  |  |
|  | **Hydroxyisocaproic acid** | HMDB00746 | 0.50/0.78 |  |  |
|  | **2-hydroxycaproic acid** | HMDB01624 | 0.86/0.94 |  |  |
|  | **(R)-3-hydroxyhexanoic acid** | HMDB10718 | 0.57/0.58 |  |  |
|  | **6-hydroxyhexanoic acid** | HMDB12843 | 0.29/0.37 |  |  |
|  | **Threo-3-hydroxy-2-methylbutyric acid** | HMDB59770 | 0.23/0.30 |  |  |
|  | **3-hydroxyhexanoic acid** | HMDB61652 | 0.57/0.58 |  |  |
| M138T392 [M-H_2_O+H]^+^  Monoisotopic mass : 155.062784  Calcd. mass : 155.069477 | **L-Histidine** | HMDB00177 | -3.10/-3.60 | 15.0 | 0.0002 |
| M138T392 [M+Na]^+^  Monoisotopic mass : 115.065675  Calcd. mass : 115.063329 | **L-Proline** | HMDB00162 | -2.70/-2.60 |  |  |
|  | **Acetamidopropanal** | HMDB12880 | -0.72/-1.30 |  |  |
| M141T484 [M+H]^+^  Monoisotopic mass : 140.058392  Calcd. mass : 140.058578 | **1,3-Dimethyluracil** | HMDB02144 | -0.81/-0.41 | 6.3 | 7.9E-10 |
|  | **Imidazolepropionic acid** | HMDB02271 | -0.27/-1.11 |  |  |
|  | **Methylimidazoleacetic acid** | HMDB02820 | -0.22/-1.20 |  |  |
|  | **Pi-Methylimidazoleacetic acid** | HMDB04988 | -0.39/-1.20 |  |  |
| M142T101 [M+H]^+^  Monoisotopic mass : 141.024743  Calcd. mass : 141.019094 | **O-Phosphoethanolamine** | HMDB00224 | -1.50/-2.50 | 4.7 | 0 |
| M142T161 [M-H_2_O+H]^+^  Monoisotopic mass : 159.105251  Calcd. mass : 159.102668 | **8-hydroxyoctanoate** | HMDB61914 | 1.58/1.26 | 3.3 | 1.3E-5 |
| M144T391 [M+H]^+^  Monoisotopic mass : 143.058059  Calcd. mass : 143.058243 | **Vinylacetylglycine** | HMDB00894 | -0.48/-0.49 | 3.8 | 1.7E-5 |
| M144T391 [M-H_2_O+H]^+^  Monoisotopic mass : 161.073339  Calcd. mass : 161.068808 | **Aminoadipic acid** | HMDB00510 | -3.40/-2.80 |  |  |
|  | **N-acetylthreonine** | HMDB62557 | -0.60/-0.57 |  |  |
|  | **N-methyl-L-glutamic acid** | HMDB62660 | -2.60/-3.0 |  |  |
| M144T402  Monoisotopic mass : 144.081263 | **No hit** | -- | -- | 2.3 | 1.5E-10 |
| M146T390 [M+H]^+^  Monoisotopic mass : 145.110085  Calcd. mass : 145.110279 | **3-dehydroxycarnitine** | HMDB06831 | -3.10/-4.00 | 2.8 | 1.4E-13 |
| M146T419  Monoisotopic mass : 146.060480 | **No hit** | -- | -- | 2.7 | 3.1E-14 |
| M149T401  Monoisotopic mass : 149.060249 | **No hit** | -- | -- | 2.4 | 2.5E-11 |
| M158T393/M158T456  [M-H_2_O+H]^+^  Monoisotopic mass : 175.125398/175.125417  Calcd. mass : 175.119501 | **L-argininium** | HMDB62762 | -0.59/-3.20 | 3.0/  2.4 | 3.3E-11/  6.2E-13 |
| M159T419  [M-H_2_O+H]^+^  Monoisotopic mass : 176.099460  Calcd. mass : 176.094963 | **Serotonin** | HMDB00259 | 0.56/0.48 | 2.8 | 5.4E-16 |
| M160T101/M160T125  [M-H_2_O+Na]^+^  Monoisotopic mass : 155.068611/155.068638  Calcd. mass : 155.069476 | **L-Histidine** | HMDB00177 | -3.10/-3.60 | 2.8/3.1 | 5.1E-14/  4.3E-13 |
| M160T288  [M-H_2_O+H]^+^  Monoisotopic mass : 177.038744  Calcd. mass : 177.045964 | **N-Formyl-L-methionine** | HMDB01015 | -0.61/-0.16 | 6.3 | 7.8E-6 |
| M162T101/M162T126  [M+H]^+^  Monoisotopic mass : 161.030946/161.030983  Calcd. mass : 161.032422 | **4-hydroxy-L-glutamic acid** | HMDB02273 | -1.50/-4.20 | 2.7/3.1 | 5.1E-14/  4.3E-13 |
|  | **N-formyl-L-aspartate** | HMDB60495 | -0.86/-0.63 |  |  |
| M166T253/M166T315  [M+H]^+^  Monoisotopic mass : 165.064916/165.064882  Calcd. mass : 165.065099 | **7-methylguanine** | HMDB00897 | -0.62/-0.37 | 2.5/  2.8 | 2.3E-17/  1.4E-16 |
|  | **3-methylguanine** | HMDB01566 | -0.90/-1.20 |  |  |
|  | **1-methylguanine** | HMDB03282 | -0.90/-1.20 |  |  |
|  | **N_2_-methylguanine** | HMDB06040 | -0.71/-0.31 |  |  |
| M166T253/M166T315  [M-H_2_O+Na]^+^  Monoisotopic mass : 161.098367/161.098333  Calcd. mass : 161.105193 | **L-Carnitine** | HMDB00062 | -0.71/-0.31 |  |  |
|  | **Malonyl-Carnitin** | HMDB62496 | -2.90/-4.90 |  |  |
| M166T401  [M+H]^+^  Monoisotopic mass : 165.078784  Calcd. mass : 165.078979 | **L-Phenylalanine** | HMDB00159 | -1.40/-1.20 | 2.4 | 2.0E-11 |
| M170T419  [M+H]^+^  Monoisotopic mass : 169.052470  Calcd. mass : 169.050394 | **Phosphodimethylethanolamine** | HMDB60244 | -0.68/-2.10 | 3.0 | 2.0E-14 |
| M173T423  Monoisotopic mass : 173.129011 | **No hit** | -- | -- | 2.3 | 8.2E-11 |
| M174T522  [M+H]^+^  Monoisotopic mass : 173.116321  Calcd. mass : 173.116427 | **Apo-[3-methylcrotonoyl-CoA:carbon-dioxide ligase (ADP-forming)]** | HMDB59607 | -1.90/-1.70 | 2.6 | 2.7E-12 |
| M177T101  Monoisotopic mass : 176.066491  Calcd. mass : 176.068473 | **2-Isopropylmalic acid** | HMDB00402 | -0.29/0.21 | 2.6 | 3.0E-10 |
|  | **2,3-Dimethyl-3-hydroxyglutaric acid** | HMDB02025 | -0.23/-0.21 |  |  |
| M183T500 [M+H]^+^  Monoisotopic mass : 182.077149  Calcd. mass : 182.079038 | **Galactitol** | HMDB00107 | -2.70/-3.70 | 2.0 | 4.0E-15 |
|  | **Sorbitol** | HMDB00247 | -2.70/-3.70 |  |  |
|  | **Mannitol** | HMDB00765 | -2.70/-3.70 |  |  |
| M183T500 [M+Na]^+^  Monoisotopic mass : 160.095320  Calcd. mass : 160.10005 | **Tryptamine** | HMDB00303 | 1.21/1.49 |  |  |
| M184T56 [M+H]^+^  Monoisotopic mass : 183.053085  Calcd. mass : 183.053158 | **4-pyridoxic acid** | HMDB00017 | -0.08/-0.75 | 2.8 | 1.3E-12 |
| M184T56 [M-H_2_O+H]^+^  Monoisotopic mass : 201.068365  Calcd. mass : 201.048197 | **N-acetyl-L-2-aminoadipate** | HMDB62715 | 0.12/0.15 |  |  |
| M184T56 [M+Na]^+^  Monoisotopic mass : 161.071256  Calcd. mass : 161.068808 | **Aminoadipic acid** | HMDB00510 | -3.40/-2.80 |  |  |
|  | **N-acetylthreonine** | HMDB62557 | -0.60/-0.57 |  |  |
|  | **N-methyl-L-glutamic acid** | HMDB62660 | -2.60/-3.00 |  |  |
| M186T391 [M-H_2_O+H]^+^  Monoisotopic mass : 203.083988  Calcd. mass : 203.090606 | **Alanyl-Asparagine** | HMDB28682 | -3.10/-4.80 | 57.6 | 0.0005 |
|  | **Asparaginyl-Alanine** | HMDB28724 | -3.20/-4.80 |  |  |
|  | **Glutaminyl-Glycine** | HMDB28797 | -3.30/-5.10 |  |  |
|  | **Glycyl-Glutamine** | HMDB28839 | -3.40/-5.10 |  |  |
|  | **Glycyl-Gamma-glutamate** | HMDB28855 | -3.30/-5.00 |  |  |
|  | **Gamma-glutamyl-Glycine** | HMDB29149 | -3.30/-5.10 |  |  |
| M187T987 [M+H]^+^  Monoisotopic mass : 185.987940  Calcd. mass : 185.992939 | **2-Phosphoglyceric acid** | HMDB00362 | -2.20/-1.60 | 3.6 | 0 |
|  | **3-Phosphoglyceric acid** | HMDB00807 | -2.30/-1.60 |  |  |
|  | **2-Phospho-D-glyceric acid** | HMDB03391 | -2.20/-1.60 |  |  |
|  | **(2R)-2-hydroxy-3-(phosphanatooxy)propanoate** | HMDB60180 | -2.30/-1.60 |  |  |
| M188T419 [M+H]^+^  Monoisotopic mass : 185.987940  Calcd. mass : 185.992939 | **Indolelactic acid** | HMDB00671 | 1.33/1.28 | 2.8 | 7.9E-14 |
|  | **5-Methoxyindoleacetate** | HMDB04096 | 1.76/1.55 |  |  |
| M189T420 [M+H]^+^  Monoisotopic mass : 188.066595  Calcd. mass : 206.090271 | **Serinyl-Threonine** | HMDB29049 | -3.10/-5.10 | 2.8 | 7.4E-14 |
|  | **Threoninyl-Serine** | HMDB29070 | -3.10/-5.10 |  |  |
| M192T150 [M+H]^+^  Monoisotopic mass : 191.055585  Calcd. mass : 191.058243 | **5-hydroxyindoleacetic acid** | HMDB00763 | 1.28/1.41 | 3.7 | 6.4E-15 |
| M192T150 [M-H_2_O+H]^+^  Monoisotopic mass : 209.070865  Calcd. mass : 209.068808 | **Hydroxyphenylacetylglycine** | HMDB00735 | 0.46/0.20 |  |  |
| M192T150 [M+Na]^+^  Monoisotopic mass : 169.073756  Calcd. mass : 169.073893 | **Norepinephrine** | HMDB00216 | -1.40/-0.68 |  |  |
|  | **Pyridoxine** | HMDB00239 | -0.57/-0.95 |  |  |
|  | **6-hydroxydopamine** | HMDB01537 | -0.91/-0.15 |  |  |
|  | **5-hydroxydopamine** | HMDB04817 | -0.83/-0.42 |  |  |
| M192T150 [M-H_2_O+Na]^+^  Monoisotopic mass : 187.089036  Calcd. mass : 187.084458 | **2-keto-6-acetamidocaproate** | HMDB12150 | -0.10/-0.07 |  |  |
| M202T165[M-H_2_O+H]^+^  Monoisotopic mass : 219.094601  Calcd. mass : 219.085521 | **Asparaginyl-Serine** | HMDB28740 | -3.50/-5.90 | 2.6 | 2.6E-14 |
|  | **Serinyl-Asparagine** | HMDB29034 | -3.50/-5.90 |  |  |
| M204T390 [M+H]^+^  Monoisotopic mass : 203.115755  Calcd. mass : 203.115758 | **N-lactoyl-Leucine** | HMDB62176 | 0.12/0.25 | 4.2 | 2.9E-12 |
|  | **L-Acetylcarnitine** | HMDB00201 | -2.40/-4.40 |  |  |
| M205T419 [M+H]^+^  Monoisotopic mass : 204.089835  Calcd. mass : 204.089878 | **L-Tryptophan** | HMDB00929 | -1.10/-1.10 | 2.8 | 3.9E-14 |
| M205T419 [M-H_2_O+H]^+^  Monoisotopic mass : 222.105115  Calcd. mass : 222.100442 | **Glycyl-Phenylalanine** | HMDB28848 | -1.10/-2.30 |  |  |
|  | **Phenylalanyl-Glycine** | HMDB28995 | -0.90/-2.30 |  |  |
| M208T181 [M+H]^+^  Monoisotopic mass : 207.089473  Calcd. mass : 207.089543 | **N-Acetyl-L-phenylalanine** | HMDB00512 | 1.11/0.90 | 3.0 | 3.7E-11 |
|  | **Phenylpropionylglycine** | HMDB00860 | 0.93/0.95 |  |  |
|  | **3-Phenylpropionylglycine** | HMDB02042 | -1.30/-1.70 |  |  |
|  | **2-Acetamido-4-methylphenyl acetate** | HMDB61682 | 1.28/1.98 |  |  |
| M208T181 [M-H_2_O+Na]^+^  Monoisotopic mass : 203.122924  Calcd. mass : 203.126991 | **Glycyl-Lysine** | HMDB28846 | -3.40/-4.20 |  |  |
|  | **Lysyl-Glycine** | HMDB28951 | -3.40/-4.20 |  |  |
| M209T384 [M+H]^+^  Monoisotopic mass : 208.048354  Calcd. mass : 208.051777 | **Cysteinyl-Serine** | HMDB28784 | -2.90/-4.40 | 14.6 | 4.6E-12 |
|  | **Serinyl-Cysteine** | HMDB29036 | -2.90/-4.40 |  |  |
| M226T325 [M-H_2_O+H]^+^  Monoisotopic mass : 243.090194  Calcd. mass : 243.085520 | **Asparaginyl-Alanine** | HMDB28724 | -3.20/-4.80 | 2.8 | 9.8E-14 |
|  | **Glutaminyl-Glycine** | HMDB28797 | -3.30/-5.10 |  |  |
|  | **Glycyl-Glutamine** | HMDB28839 | -3.40/-5.10 |  |  |
|  | **Glycyl-Gamma-glutamate** | HMDB28855 | -3.30/-5.00 |  |  |
|  | **Gamma-glutamyl glycine** | HMDB29149 | -3.30/-5.10 |  |  |
| M227T103 [M-H_2_O+H]^+^  Monoisotopic mass : 244.182764  Calcd. mass : 244.178692 | **Isoleucyl-Isoleucine** | HMDB28910 | -1.00/-0.72 | 4.8 | 2.1E-5 |
|  | **Isoleucyl-Leucine** | HMDB28911 | -0.96/-0.79 |  |  |
|  | **Leucyl-Isoleucine** | HMDB28932 | -0.89/-0.79 |  |  |
|  | **Leucyl-Leucine** | HMDB28933 | -0.86/-0.87 |  |  |
| M232T466 [M+H]^+^  Monoisotopic mass : 231.147007  Calcd. mass : 231.147058 | **O-butanoyl-carnitine** | HMDB62510 | -2.10/-3.30 | 2.5 | 7.5E-12 |
|  | **O-isobutyryl-L-carnitine** | HMDB62606 | -2.10/-3.20 |  |  |
|  | **Isobutyryl-L-carnitine** | HMDB00736 | -2.10/-3.20 |  |  |
|  | **Butyrylcarnitine** | HMDB02013 | -2.10/-3.30 |  |  |
| M245T384 [M+H]^+^  Monoisotopic mass : 244.069590  Calcd. mass : 244.069536 | **Uridine** | HMDB00296 | -1.80/-2.40 | 17.4 | 2.9E-15 |
|  | **Pseudouridine** | HMDB00767 | -2.00/-3.10 |  |  |
| M245T384 [M-H_2_O+H]^+^  Monoisotopic mass : 262.084870  Calcd. mass : 262.080100 | **L-beta-aspartyl-L-glutamic acid** | HMDB11164 | -3.30/-4.50 |  |  |
|  | **Na-L-Glutamyl-L-aspartic acid** | HMDB30413 | -3.20/-3.90 |  |  |
|  | **Ng-L-Glutamyl-L-aspartic acid** | HMDB30419 | -3.20/-4.50 |  |  |
| M245T401  Monoisotopic mass : 244.650557 | **No hit** | -- | -- | 2.4 | 2.4E-11 |
| M246T444 [M+H]^+^  Monoisotopic mass : 245.162715  Calcd. mass : 245.162708 | **Isovalerylcarnitine** | HMDB00378 | -1.90/-3.00 | 3.1 | 1.3E-15 |
|  | **Valerylcarnitine** | HMDB13128 | -1.90/-2.90 |  |  |
|  | **Pivaloylcarnitine** | HMDB41993 | -2.00/-2.60 |  |  |
| M249T420 [M+H]^+^  Monoisotopic mass : 248.053750  Calcd. mass : 248.056208 | **Pyridoxamine 5’-phosphate** | HMDB01555 | -0.99/-2.20 | 2.7 | 3.0E-13 |
| M249T420 [M-H_2_O+Na]^+^  Monoisotopic mass : 244.087201  Calcd. mass : 244.088163 | **Biotin** | HMDB00030 | 0.17/0.32 |  |  |
| M251T258 [M+H]^+^  Monoisotopic mass : 250.070548  Calcd. mass : 250.062342 | **Gamma-Glutamylcysteine** | HMDB01049 | -2.50/-3.80 | 2.7 | 2.0E-13 |
| M251T258 [M-H_2_O+H]^+^  Monoisotopic mass : 268.085828  Calcd. mass : 268.080770 | **Inosine** | HMDB00195 | -1.70/-2.00 |  |  |
| M251T258 [M-H_2_O+H]^+^  Monoisotopic mass : 268.085828  Calcd. mass : 268.088163 | **Cysteinyl-Phenylalanine** | HMDB28782 | -1.30/-1.70 |  |  |
|  | **Phenylalanyl-Cysteine** | HMDB28992 | -1.30/-1.70 |  |  |
| M251T258 [M-H_2_O+Na]^+^  Monoisotopic mass : 246.103999  Calcd. mass : 246.103813 | **Methionyl-Proline** | HMDB28981 | -0.55/-2.50 |  |  |
|  | **Prolyl-Methionine** | HMDB29023 | -1.10/-2.50 |  |  |
| M257T248 [M+H]^+^  Monoisotopic mass : 256.101159  Calcd. mass : 256.105922 | **1-(beta-D-Ribofuranosyl)-1,4-dihydronicotinamide** | HMDB11648 | -1.90/-2.10 | 2.6 | 1.7E-12 |
| M257T248 [M-H_2_O+H]^+^  Monoisotopic mass : 274.121249  Calcd. mass : 274.127720 | **Glutaminyl-Glutamine** | HMDB28795 | -3.50/-5.70 |  |  |
|  | **Glutaminyl-Gamma-glutamate** | HMDB2881 | -3.40/-5.60 |  |  |
|  | **Gamma-glutamyl-Glutamine** | HMDB29147 | -3.50/-5.70 |  |  |
|  | **Gamma-glutamyl-Gamma-glutamate** | HMDB29163 | -3.40/-5.60 |  |  |
| M257T248 [M-H_2_O+Na]^+^  Monoisotopic mass : 252.139420  Calcd. mass : 252.136159 | **QH(2)** | HMDB59661 | 2.70/3.29 |  |  |
| M258T288 [M+H]^+^  Monoisotopic mass : 257.101159  Calcd. mass : 257.101171 | **5-Methylcytidine** | HMDB00982 | -2.20/-2.40 | 3.5 | 5.6E-12 |
| M258T646 [M+H]^+^  Monoisotopic mass : 257.101159  Calcd. mass : 257.101171 | **Glycerophosphocholine** | HMDB00086 | -2.60/-5.70 | 9.7 | 2.5E-9 |
| M269T988 [M+Na]^+^  Monoisotopic mass : 246.009266  Calcd. mass : 246.005826 | **Dimethylallylpyrophosphate** | HMDB01120 | 0.30/0.30 | 4.0 | 0 |
|  | **Isopentenyl pyrophosphate** | HMDB01347 | 0.04/0.20 |  |  |
| M273T258 [M-H_2_O+Na]^+^  Monoisotopic mass : 268.085662  Calcd. mass : 268.080770 | **Inosine** | HMDB00195 | -1.70/-2.00 | 3.3 | 1.0E-13 |
| M277T335 [M+H]^+^  Monoisotopic mass : 276.110983  Calcd. mass : 276.106984 | **5-amino-6-ribitylamino uracil** | HMDB11106 | -2.40/-4.40 | 3.2 | 6.0E-15 |
| M277T335 [M+H]^+^  Monoisotopic mass : 276.110983  Calcd. mass : 276.111001 | **N-lactoyl-Tryptophan** | HMDB62176 | 0.12/0.25 |  |  |
| M277T335 [M-H_2_O+H]^+^  Monoisotopic mass : 294.126263  Calcd. mass : 294.121572 | **Glutamylphenylalanine** | HMDB00594 | -2.10/-2.10 |  |  |
|  | **Hydroxyprolyl-Tyrosine** | HMDB28875 | -2.00/-2.90 |  |  |
|  | **Tyrosyl-Hydroxyproline** | HMDB29106 | -2.20/-2.90 |  |  |
|  | **N-gamma-L-glutamyl-L-phenylalanine** | HMDB29562 | -2.00/-1.30 |  |  |
| M280T646 [M-H_2_O+H]^+^  Monoisotopic mass : 297.100153  Calcd. mass : 297.107319 | **1-Methylguanosine** | HMDB01563 | -1.80/-2.50 | 8.7 | 2.7E-8 |
|  | **2-Methylguanosine** | HMDB05862 | -1.50/-1.90 |  |  |
|  | **3’-O-Methylguanosine** | HMDB06038 | -1.10/-1.50 |  |  |
| M280T646 [M+Na]^+^  Monoisotopic mass : 257.103044  Calcd. mass : 257.101171 | **5-Methylcytidine** | HMDB00982 | -2.20/-2.40 |  |  |
| M280T646 [M+Na]^+^  Monoisotopic mass : 257.103044  Calcd. mass : 257.102824 | **Glycerophosphocholine** | HMDB00086 | -2.60/-5.70 |  |  |
| M285T968  Monoisotopic mass : 284.972997 | **No hit** | -- | -- | 3.5 | 0 |
| M298T341 [M+H]^+^  Monoisotopic mass : 297.107656  Calcd. mass : 297.107319 | **1-Methylguanosine** | HMDB01563 | -1.80/-2.50 | 6.9 | 2.4E-8 |
|  | **2-Methylguanosine** | HMDB05862 | -1.50/-1.90 |  |  |
|  | **3’-O-Methylguanosine** | HMDB06038 | -1.10/-1.50 |  |  |
| M298T341 [M+Na]^+^  Monoisotopic mass : 275.125827  Calcd. mass : 275.126991 | **Alanyltryptophan** | HMDB13209 | -2.80/-5.40 |  |  |
|  | **Tryptophyl-Alanine** | HMDB29076 | -0.88/-1.60 |  |  |
| M298T341 [M-H_2_O+Na]^+^  Monoisotopic mass : 293.141107  Calcd. mass : 293.137556 | **Glutaminyl-Phenylalanine** | HMDB28804 | -2.50/-2.90 |  |  |
|  | **Phenylalanyl-Glutamine** | HMDB28993 | -2.40/-2.90 |  |  |
|  | **Phenylalanyl-Gamma-glutamate** | HMDB29009 | -1.60/-2.70 |  |  |
|  | **Gamma-glutamyl-Phenylalanine** | HMDB29156 | -2.50/-2.90 |  |  |
| M326T353  Monoisotopic mass : 326.378822 | **No hit** | -- | -- | 2.8 | 7.2E-12 |
| M335T127/M335T379  [M+Na]^+^  Monoisotopic mass : 312.116953/312.116958  Calcd. mass : 312.114378 | **Methionyl-Tyrosine** | HMDB28985 | -0.56/-1.40 | 2.8 | 4.9E-13 |
|  | **Tyrosyl-Methionine** | HMDB29111 | -0.56/-1.40 |  |  |
| M338T378 [M+H]^+^  Monoisotopic mass : 337.096947  Calcd. mass : 337.094371 | **S-(Hydroxymethyl)glutathione** | HMDB04662 | -3.20/-5.50 | 2.9 | 5.2E-13 |
| M340T357 [M+H]^+^  Monoisotopic mass : 339.350412  Calcd. mass : 339.350115 | **Docosanamide** | HMDB00583 | 9.15/8.12 | 9.2 | 1.9E-13 |
| M342T360  Monoisotopic mass : 342.373728 | **No hit** | -- | -- | 11.2 | 2.2E-9 |
| M355T335 [M+Na]^+^  Monoisotopic mass : 332.123198  Calcd. mass : 332.124646 | **Glutamyl-Tryptophan** | HMDB28830 | -1.20/-2.20 | 3.4 | 1.1E-13 |
|  | **Tryptophyl-Glutamate** | HMDB29082 | -1.10/-2.20 |  |  |
|  | **Gamma-glutamyl-Phenylalanine** | HMDB29156 | -2.50/-2.90 |  |  |
| M367T96 [M+Na]^+^  Monoisotopic mass : 344.143266  Calcd. mass : 344.137222 | **Tyrosyl-Tyrosine** | HMDB29117 | -1.30/-0.68 | 4.5 | 3.8E-10 |
| M367T967  Monoisotopic mass : 366.976477 | **No hit** | -- | -- | 3.9 | 0 |
| M377T306 [M+H]^+^  Monoisotopic mass : 376.138497  Calcd. mass : 376.138284 | **Riboflavin** | HMDB00244 | -1.10/-0.92 | 3.5 | 4.4E-16 |
| M399T306 [M+H]^+^  Monoisotopic mass : 376.138497  Calcd. mass : 376.138284 | **Riboflavin** | HMDB00244 | -1.10/-0.92 | 3.5 | 8.8E-16 |
| M422T311  Monoisotopic mass : 422.186701 | **No hit** | -- | -- | 2.9 | 6.8E-14 |
| M431T284 [M-H_2_O+H]^+^  Monoisotopic mass : 426.145568  Calcd. mass : 426.148574 | **Chitobiose** | HMDB03556 | -2.90/-4.80 | 7.1 | 3.0E-7 |
| M433T989  Monoisotopic mass : 433.005819 | **No hit** | -- | -- | 7.1 | 0 |
| M449T966  Monoisotopic mass : 448.979723 | **No hit** | -- | -- | 4.8 | 0 |
| M453T285  Monoisotopic mass : 453.102104 | **No hit** | -- | -- | 6.2 | 2.4E-8 |
| M454T373  Monoisotopic mass : 454.111554 | **No hit** | -- | -- | 3.0 | 6.9E-15 |
| M465T964  Monoisotopic mass : 464.952486 | **No hit** | -- | -- | 16.0 | 6.1E-14 |
| M487T59  Monoisotopic mass : 487.193204 | **No hit** | -- | -- | 3.7 | 2.0E-13 |
| M515T348  Monoisotopic mass : 515.171220 | **No hit** | -- | -- | 3.4 | 4.0E-15 |
| M515T953  Monoisotopic mass : 515.009318 | **No hit** | -- | -- | 2.4 | 3.5E-11 |
| M528T336 [M-H_2_O+H]^+^  Monoisotopic mass : 545.188906  Calcd. mass : 545.195584 | **Lacto-N-triaose** | HMDB06592 | -2.70/-7.40 | 2.4 | 3.2E-11 |
|  | **Lacto-N-triose I** | HMDB39750 | -2.60/-6.80 |  |  |
| M528T366  Monoisotopic mass : 528.488754 | **No hit** | -- | -- | 3.0 | 1.1E-14 |
| M531T965  Monoisotopic mass : 530.983242 | **No hit** | -- | -- | 5.6 | 0 |
| M537T348  Monoisotopic mass : 537.153006 | **No hit** | -- | -- | 6.2 | 8.0E-15 |
| M542T58  Monoisotopic mass : 542.245112 | **No hit** | -- | -- | 3.2 | 8.9E-16 |
| M545T333  Monoisotopic mass : 545.208014 | **No hit** | -- | -- | 2.6 | 8.6E-13 |
| M550T336  Monoisotopic mass : 542.245112 | **No hit** | -- | -- | 2.2 | 9.2E-10 |
| M586T333  Monoisotopic mass : 586.234827 | **No hit** | -- | -- | 4.1 | 0 |
| M669T125  Monoisotopic mass : 669.206776 | **No hit** | -- | -- | 10.4 | 1.4E-10 |
| M669T316  Monoisotopic mass : 669.206078 | **No hit** | -- | -- | 5.51 | 0 |
| M679T953  Monoisotopic mass : 679.015879 | **No hit** | -- | -- | 3.0 | 9.3E-15 |
| M691T100  Monoisotopic mass : 691.188534 | **No hit** | -- | -- | 4.0 | 2.2E-15 |
| M691T125  Monoisotopic mass : 691.188692 | **No hit** | -- | -- | 5.1 | 1.9E-10 |
| M761T953  Monoisotopic mass : 761.019256 | **No hit** | -- | -- | 3.2 | 3.5E-15 |
| M843T953  Monoisotopic mass : 702.976169 | **No hit** | -- | -- | 3.9 | 0 |

**Table S8** Tentative identification of dysregulated metabolites upon MMW exposure in negative-ion mode exocellular metabolomics.

| Feature of interest | **HMDB hits (±0.003 Da)** | HMDB ID | LogP | FC | p-value |
| --- | --- | --- | --- | --- | --- |
| M74T101  Monoisotopic mass : 74.000600 | **No hit** | -- | -- |  |  |
| M79T167  Monoisotopic mass : 78.918900 | **No hit** | -- | -- |  |  |
| M80T92  Monoisotopic mass : 79.957660 | **No hit** | -- | -- |  |  |
| M87T70 [M-H]^-^  Monoisotopic mass : 88.016700  Calcd. mass : 88.016044 | **2-hydroxyacrylic acid** | HMDB62676 | -0.09/-0.01 |  |  |
|  | **Pyruvic acid** | HMDB00243 | -0.38/0.07 |  |  |
|  | **Malonic semialdehyde** | HMDB11111 | -0.69/-0.49 |  |  |
| M100T438 [M-H_2_O-H]^-^  Monoisotopic mass : 119.063677  Calcd. mass : 119.058243 | **L-Threonine** | HMDB00167 | -3.00/-3.50 | 2.2 | 1.5E-9 |
|  | **L-Homoserine** | HMDB00719 | -3.30/-3.80 |  |  |
|  | **L-Allothreonine** | HMDB04041 | -3.00/-3.50 |  |  |
|  | **4-amino-3-hydroxybutyrate** | HMDB61877 | -3.40/-3.80 |  |  |
| M101T118 [M-H]^-^  Monoisotopic mass :  102.032408  Calcd. mass : 102.031694 | **2-ketobutyric acid** | HMDB00005 | 0.07/0.77 | 7.8 | 8.9E-6 |
|  | **Acetoacetic acid** | HMDB00060 | -0.47/0.00 |  |  |
|  | **2-Methyl-3-oxopropanoic acid** | HMDB01172 | 0.00/0.05 |  |  |
|  | **Succinic acid semialdehyde** | HMDB01259 | -0.47/-0.56 |  |  |
|  | **(S)-Methylmalonic acid semialdehyde** | HMDB02217 | 0.00/0.05 |  |  |
| M101T118 [M-H_2_O-H]^-^  Monoisotopic mass : 120.047688  Calcd. mass : 120.042259 | **(S)-3,4-Dihydroxybutyric acid** | HMDB00337 | -1.50/-1.40 |  |  |
|  | **4-Deoxyerythronic acid** | HMDB00498 | -1.20/-1.10 |  |  |
|  | **4-Deoxythreonic acid** | HMDB02453 | -1.20/-1.10 |  |  |
|  | **A,b-Dihydroxyisobutyric acid** | HMDB02601 | -1.20/-1.10 |  |  |
|  | **Erythrose** | HMDB02649 | -2.30/-1.70 |  |  |
|  | **L-Erythrulose** | HMDB06293 | -2.00/-2.00 |  |  |
| M111T133 [M-H]^-^  Monoisotopic mass :  112.028011  Calcd. mass : 112.027277 | **Uracil** | HMDB00300 | -1.20/-0.86 | 3.3 | 3.2E-7 |
| M114T405 [M-H]^-^  Monoisotopic mass :  115.038955  Calcd. mass : 115.039519 | **3-keto-2-Methylbutyrate** | HMDB29172 | 0.03/0.54 | 2.9 | 6.3E-14 |
| M126T390 [M-H_2_O-H]^-^  Monoisotopic mass :  145.042997  Calcd. mass : 145.037508 | **2-keto-glutaramic acid** | HMDB01552 | -1.40/-0.92 | 8.1 | 0.00018 |
| M129T382  Monoisotopic mass :  128.892248 | **No hit** | -- | -- | 4.2 | 5.1E-11 |
| M130T391/M130T412/  M130T434 [M-H]^-^  Monoisotopic masses :  131.095310/131.095298/131.095299  Calcd. mass : 131.094629 | **L-Isoleucine** | HMDB00172 | -1.70/-1.50 | 2.1/  2.2/  2.3 | 3.3E-12/  8.7E-10/  1.5E-10 |
|  | **L-Alloisoleucine** | HMDB00557 | -1.70/-1.50 |  |  |
|  | **L-Leucine** | HMDB00687 | -1.80/-1.60 |  |  |
|  | **Beta-Leucine** | HMDB03640 | -2.10/-1.90 |  |  |
|  | **N-methylvaline** | HMDB61716 | -1.80/-1.70 |  |  |
| M131T418 [M-H]^-^  Monoisotopic mass :  132.098598  Calcd. mass : 132.089878 | **Ornithine** | HMDB00214 |  | 2.2 | 7.3E-10 |
| M137T257 [M-H]^-^  Monoisotopic mass :  138.032451  Calcd. mass : 138.031694 | **4-hydroxybenzoic acid** | HMDB00500 | 1.58/1.33 | 2.4 | 1.5E-10 |
|  | **3-hydroxybenzoic acid** | HMDB02466 | 1.81/1.33 |  |  |
|  | **Keratan sulfate II (core 2-linked), degradation product 1** | HMDB62484 | 0.90/1.73 |  |  |
|  | **Gentisate aldehyde** | HMDB04062 | 1.02/1.73 |  |  |
| M137T257 [M-H_2_O-H]^-^  Monoisotopic mass :  156.047731  Calcd. mass : 156.042259 | **2,3-Methyleneglutaric acid** | HMDB59731 | 0.77/0.44 |  |  |
| M141T988 [M-H_2_O-H]^-^  Monoisotopic mass :  160.040254  Calcd. mass : 160.037173 | **Oxoadipic acid** | HMDB00225 | -0.37/0.34 | 2.7 | 8.4E-15 |
|  | **3-Oxoadipic acid** | HMDB00398 | -0.64/-0.18 |  |  |
| M149T439 [M-H]^-^  Monoisotopic mass :  150.055174  Calcd. mass : 150.052823 | **D-Xylose** | HMDB00098 | -2.60/-2.30 | 2.1 | 3.2E-5 |
|  | **D-Ribose** | HMDB00283 | -2.60/-2.30 |  |  |
|  | **2-Deoxyribonic acid** | HMDB00366 | -2.00/-2.10 |  |  |
|  | **D-Ribulose** | HMDB00621 | -2.20/-2.60 |  |  |
|  | **Arabinofuranose** | HMDB12325 | -2.60/-2.30 |  |  |
|  | **2-Deoxypentonic acid** | HMDB59753 | -2.00/-2.10 |  |  |
|  | **Aldehydo-D-xylose** | HMDB60254 | -2.30/-2.90 |  |  |
|  | **Beta-D-ribopyranose** | HMDB12194 | -2.60/-2.30 |  |  |
| M151T304 [M-H]^-^  Monoisotopic mass :  152.034151  Calcd. mass : 152.033425 | **Xanthine** | HMDB00292 | -0.65/-0.21 | 3.5 | 5.0E-8 |
|  | **6,8-dihydroxypurine** | HMDB01182 | -1.00/-1.60 |  |  |
| M157T55 [M-H]^-^  Monoisotopic mass :  158.058602  Calcd. mass : 158.057909 | **Succinylacetone** | HMDB00635 | -0.18/0.15 | 2.7 | 0.00065 |
|  | **Isopropylmaleate** | HMDB12241 | 1.05/1.09 |  |  |
| M157T55 [M-H_2_O-H]^-^  Monoisotopic mass :  176.073882  Calcd. mass : 176.068435 | **2-Isopropylmalic acid** | HMDB00402 | -0.29/0.21 |  |  |
|  | **3-Isopropylmalate** | HMDB12156 | 0.28/0.16 |  |  |
|  | **3-Hydroxy-2-methylglutarate** | HMDB29169 | -0.30/-0.04 |  |  |
| M157T313 [M-H]^-^  Monoisotopic mass :  158.035291  Calcd. mass : 158.032757 | **4,5-Dihydroorotic acid** | HMDB00528 | -1.70/-1.50 | 3.4 | 8.4E-15 |
|  | **L-Dihydroorotic acid** | HMDB03349 | -1.70/-1.50 |  |  |
| M157T405 [M-H]^-^  Monoisotopic mass :  158.044701  Calcd. mass : 158.043990 | **Allantoin** | HMDB00462 | -1.90/-2.40 | 2.6 | 2.5E-13 |
| M157T405 [M-H_2_O-H]^-^  Monoisotopic mass :  176.059981  Calcd. mass : 176.054555 | **Allantoic acid** | HMDB01209 | -2.10/-2.60 |  |  |
| M159T313 [M-H_2_O-H]^-^  Monoisotopic mass :  178.047638  Calcd. mass : 178.041213 | **Cysteinylglycine** | HMDB00078 | -2.60/-3.90 | 3.5 | 7.3E-15 |
|  | **Cysteinyl-Glycine** | HMDB28775 | -2.60/-3.90 |  |  |
|  | **Glycyl-Cysteine** | HMDB28838 | -2.60/-3.90 |  |  |
| M159T1022  [M-H_2_O+HCOOH-H]^-^  Monoisotopic mass :  131.986958  Calcd. mass : 132.00587 | **Oxalacetic acid** | HMDB00223 | -0.68/-0.04 | 4.2 | 8.5E-14 |
| M163T77 [M-H]^-^  Monoisotopic mass :  164.048125  Calcd. mass : 164.047344 | **Phenylpyruvic acid** | HMDB00205 | 1.30/1.90 | 92.3 | 0.00043 |
|  | **Enol-phenylpyruvate** | HMDB12225 | 1.55/1.62 |  |  |
| M179T273 [M-H]^-^  Monoisotopic mass :  180.062411  Calcd. mass : 180.053492 | **Nicotinuric acid** | HMDB03269 | -0.23/-0.15 | 2.1 | 1.2E-7 |
|  | **Picolinoylglycine** | HMDB59766 | -0.06/-0.31 |  |  |
|  | **Isonicotinylglycine** | HMDB41912 | -0.24/-1.40 |  |  |
| M181T348 [M-H_2_O-H]^-^  Monoisotopic mass :  200.074014  Calcd. mass : 200.068473 | **(Z)-3-(1-Formyl-1-propenyl)pentanedioic acid** | HMDB33091 | 0.69/0.14 | 3.5 | 0 |
| M185T59 [M-H_2_O+HCOOH-H]^-^  Monoisotopic mass :  158.025707  Calcd. mass : 158.021523 | **2-Maleylacetate** | HMDB60348 | -0.06/0.18 | 2.1 | 1.6E-12 |
| M187T392  [M-H_2_O+HCOOH-H]^-^  Monoisotopic mass :  160.035825  Calcd. mass : 160.037173 | **Oxoadipic acid** | HMDB00225 | -0.37/0.34 | 17.2 | 7.2E-5 |
|  | **3-Oxoadipic acid** | HMDB00398 | -0.64/-0.18 |  |  |
| M187T412 [M-H_2_O-H]^-^  Monoisotopic mass :  207.069280  Calcd. mass : 207.075155 | **Dihydrolipoamide** | HMDB00985 | 2.22/1.44 | 3.2 | 0 |
| M188T424  Monoisotopic mass :  188.046039 | **No hit** | -- | -- | 2.7 | 1.5E-12 |
| M190T416 [M-H]^-^  Monoisotopic mass :  191.051094  Calcd. mass : 191.042987 | **Gamma-Carboxyglutamic acid** | HMDB41900 | -3.00/-3.60 | 4.3 | 0 |
| M190T413 [M-H_2_O-H]^-^  Monoisotopic mass :  209.066374  Calcd. mass : 209.068808 | **Hydroxyphenylacetyl**  **glycine** | HMDB00735 | 0.46/0.20 | 4.2 | 0 |
| M192T101 [M-H_2_O-H]^-^  Monoisotopic mass :  211.070539  Calcd. mass : 211.070539 | **6-carboxy-5,6,7,8-tetrahydropterin** | HMDB60410 | -1.90/-3.00 | 2.5 | 7.1E-12 |
| M192T292 [M-H]^-^  Monoisotopic mass :  193.074792  Calcd. mass : 193.073893 | **Phenylacetylglycine** | HMDB00821 | 0.51/0.51 | 2.8 | 3.4E-14 |
|  | **Methyl hippurate** | HMDB00859 | 0.96/0.67 |  |  |
|  | **2-Methylhippuric acid** | HMDB11723 | 0.66/1.04 |  |  |
| M192T292  [M-H_2_O+HCOOH-H]^-^  Monoisotopic mass :  165.064692  Calcd. mass : 165.065060 | **7-Methylguanine** | HMDB00897 | -0.62/-0.37 |  |  |
|  | **3-Methylguanine** | HMDB01566 | -0.90/-1.20 |  |  |
|  | **3-hydroxyhippuric acid** | HMDB06116 | 0.52/0.22 |  |  |
|  | **4-hydroxyhippuric acid** | HMDB13678 | 0.44/0.22 |  |  |
| M194T101/M194T127  [M-H_2_O+HCOOH-H]^-^  Monoisotopic masses :  167.042617/167.042622  Calcd. mass : 167.044324 | **2,8-Dihydroxyadenine** | HMDB00401 | -0.70/0.18 | 2.5/2.9 | 1.1E-11/  6.2E-13 |
|  | **8-Hydroxyguanine** | HMDB02032 | -1.60/-1.50 |  |  |
| M196T384  [M-H_2_O+HCOOH-H]^-^  Monoisotopic mass :  167.042617  Calcd. mass : 167.073893 | **Pyridoxine** | HMDB00239 | -0.57/-0.95 | 2.9 | 2.6E-11 |
| M201T316 [M-H_2_O-H]^-^  Monoisotopic mass :  220.077031  Calcd. mass : 220.084792 | **5-hydroxy-L-tryptophan** | HMDB00472 | -1.60/-1.40 | 2.6 | 3.0E-12 |
|  | **Oxitriptan** | HMDB15571 | -1.60/-1.40 |  |  |
| M201T316  [M-H_2_O+HCOOH-H]^-^  Monoisotopic mass :  174.051651  Calcd. mass : 174.052823 | **2,6-dimethyl-trans-2-heptenoyl-CoA** | HMDB62194 | -0.20/0.18 |  |  |
|  | **4(R),8-dimethyl-trans-2-nonenoyl-CoA** | HMDB62377 | -0.20/0.18 |  |  |
|  | **2-Isopropyl-3-oxosuccinate** | HMDB12149 | 0.43/1.23 |  |  |
|  | **Dimethyl-2-oxoglutarate** | HMDB61388 | -0.06/0.42 |  |  |
| M202T391 [M-H]^-^  Monoisotopic mass :  203.116705  Calcd. mass : 203.115758 | **N-lactoyl-leucine** | HMDB62176 | 0.12/0.25 | 3.1 | 5.0E-12 |
|  | **L-Acetylcarnitine** | HMDB00201 | -2.40/-4.40 |  |  |
| M203T419 [M-H]^-^  Monoisotopic mass :  204.090776  Calcd. mass : 204.089878 | **L-Tryptophan** | HMDB00929 | -1.10/-1.10 | 2.5 | 2.4E-12 |
| M203T419 [M-H_2_O-H]^-^  Monoisotopic mass :  222.106056  Calcd. mass : 222.100442 | **Glycyl-Phenylalanine** | HMDB28848 | -1.10/-1.23 |  |  |
|  | **Phenylalanyl-Glycine** | HMDB28995 | -0.90/-2.30 |  |  |
| M203T419  [M-H_2_O+HCOOH-H]^-^  Monoisotopic mass :  176.080676  Calcd. mass : 176.079707 | **Alanyl-Serine** | HMDB28696 | -3.10/-4.40 |  |  |
|  | **Glycyl-Threonine** | HMDB28851 | -3.00/-4.60 |  |  |
|  | **Serinyl-Alanine** | HMDB29032 | -3.10/-4.40 |  |  |
|  | **Threoninyl-Glycine** | HMDB29061 | -3.00/-4.60 |  |  |
| M206T438  Monoisotopic mass :  206.002551 | **No hit** | -- | -- | 2.6 | 1.8E-12 |
| M208T438 [M-H_2_O-H]^-^  Monoisotopic mass :  227.022726  Calcd. mass : 227.019488 | **L-Glutamic acid 5-phosphate** | HMDB01228 | -2.00/-3.00 | 2.6 | 1.6E-12 |
| M212T423 [M-H_2_O-H]^-^  Monoisotopic mass :  231.113863  Calcd. mass : 231.110673 | **Isovalerylglutamic acid** | HMDB00726 | 0.27/0.32 | 2.0 | 1.7E-7 |
|  | **Suberylglycine** | HMDB00953 | 0.32/0.27 |  |  |
| M212T423 [M-H_2_O-H]^-^  Monoisotopic mass :  231.113863  Calcd. mass : 231.121909 | **Asparaginyl-Valine** | HMDB28744 | -2.90/-3.90 |  |  |
|  | **Valyl-Asparagine** | HMDB29122 | -2.90/-3.90 |  |  |
| M215T410 [M-H_2_O-H]^-^  Monoisotopic mass :  234.05624  Calcd. mass : 234.067428 | **Cysteinyl-Hydroxyproline** | HMDB28776 | -2.90/-3.90 | 3.9 | 0 |
| M215T414 [M-H]^-^  Monoisotopic mass :  216.117312  Calcd. mass : 216.111007 | **Prolyl-Threonine** | HMDB29027 | -2.80/-3.70 | 3.3 | 2.8E-6 |
|  | **Threoninyl-Proline** | HMDB29069 | -2.60/-3.70 |  |  |
| M217T392 [M-H]^-^  Monoisotopic mass :  218.091067  Calcd. mass : 218.090272 | **Glutamylalanine** | HMDB03764 | -3.40/-4.00 | 18.2 | 6.3E-9 |
|  | **5-L-Glutamyl-L-alanine** | HMDB06248 | -3.40/-3.80 |  |  |
|  | **Hydroxyprolyl-Serine** | HMDB28872 | -3.60/-5.30 |  |  |
|  | **Serinyl-Hydroxyproline** | HMDB29040 | -3.30/-5.30 |  |  |
| M217T392  [M-H_2_O+HCOOH-H]^-^  Monoisotopic mass :  190.080967  Calcd. mass : 190.084124 | **3-Hydroxysuberic acid** | HMDB00325 | -0.17/0.15 |  |  |
|  | **6-(2-Hydroxy)-6-oxohexanoic acid** | HMDB61681 | -0.15/-0.05 |  |  |
| M217T410 [M-H_2_O-H]^-^  Monoisotopic mass :  236.053044  Calcd. mass : 236.046692 | **Aspartyl-Cysteine** | HMDB28750 | -2.70/-4.30 | 3.3 | 2.5E-14 |
|  | **Cysteinyl-Aspartate** | HMDB28771 | -2.70/-4.10 |  |  |
| M241T346 [M-H_2_O-H]^-^  Monoisotopic mass :  260.142892  Calcd. mass : 260.137222 | **L-gamma-glutamyl-L-isoleucine** | HMDB11170 | -2.60/-2.50 | 3.1 | 0 |
|  | **L-gamma-glutamyl-L-leucine** | HMDB11171 | -2.60/-2.50 |  |  |
| M241T346 [M-H_2_O-H]^-^  Monoisotopic mass :  260.142892  Calcd. mass : 260.148455 | **Asparaginyl-Lysine** | HMDB28736 | -3.50/-5.00 |  |  |
|  | **Lysyl-Asparagine** | HMDB28946 | -3.50/-5.10 |  |  |
| M243T384 [M-H]^-^  Monoisotopic mass :  244.070268  Calcd. mass : 244.069536 | **Uridine** | HMDB00296 | -1.80/-2.40 | 3.1 | 5.6E-15 |
|  | **Pseudouridine** | HMDB00767 | -2.00/-3.10 |  |  |
| M243T384 [M-H_2_O-H]^-^  Monoisotopic mass :  262.085548  Calcd. mass : 262.080101 | **L-beta-aspartyl-L-glutamic acid** | HMDB11164 | -3.30/-4.50 |  |  |
| M245T390 [M-H]^-^  Monoisotopic mass :  246.120041  Calcd. mass : 246.121572 | **L-beta-aspartyl-L-leucine** | HMDB11166 | -2.80/-2.80 | 7.5 | 0 |
|  | **L-gamma-glutamyl-L-valine** | HMDB11172 | -2.90/-2.90 |  |  |
|  | **Aspartyl-Isoleucine** | HMDB28756 | -2.80/-3.00 |  |  |
|  | **Aspartyl-Leucine** | HMDB28757 | -2.80/-3.10 |  |  |
|  | **Isoleucyl-Aspartate** | HMDB28903 | -2.70/-2.80 |  |  |
|  | **Leucyl-Aspartate** | HMDB28925 | -2.80/-2.90 |  |  |
|  | **Glutamyl-Valine** | HMDB59717 | -3.00/-3.10 |  |  |
| M255T100 [M-H]^-^  Monoisotopic mass :  256.121903  Calcd. mass : 256.117155 | **2-(3-Carboxy-3-alinopropyl)-L-histidine** | HMDB11655 | -4.10/-6.50 | 3.4 | 0 |
|  | **Histidinyl-Threonine** | HMDB28895 | -2.80/-4.50 |  |  |
|  | **Threoninyl-Histidine** | HMDB29063 | -2.90/-4.50 |  |  |
| M255T100 [M-H_2_O-H]^-^  Monoisotopic mass :  274.137183  Calcd. mass : 274.102958 | **Glutaminyl-Lysine** | HMDB28824 | -3.30/-6.10 |  |  |
|  | **Lysyl-Glutamate** | HMDB28950 | -3.20/-6.10 |  |  |
| M255T100  [M-H_2_O+HCOOH-H]^-^  Monoisotopic mass :  228.111803  Calcd. mass : 228.111007 | **Prolylhydroxyproline** | HMDB06695 | -3.00/-4.00 |  |  |
|  | **Hydroxyprolyl-Proline** | HMDB28871 | -3.00/-4.00 |  |  |
| M255T461 [M-H_2_O-H]^-^  Monoisotopic mass :  274.156193  Calcd. mass : 274.164105 | **Glutaminyl-Lysine** | HMDB28802 | -3.50/-4.80 | 2.5 | 1.7E-11 |
|  | **Lysyl-Glutamine** | HMDB28949 | -3.40/-4.80 |  |  |
|  | **Lysyl-Gamma-glutamate** | HMDB28965 | -3.30/-4.70 |  |  |
|  | **Gamma-glutamyl-Lysine** | HMDB29154 | -3.50/-4.80 |  |  |
| M256T151 [M-H_2_O-H]^-^  Monoisotopic mass :  275.106179  Calcd. mass : 275.111735 | **Norophthalmic acid** | HMDB05766 | -3.20/-4.90 | 2.8 | 1.1E-13 |
|  | **Gamma-Glutamyl Glutamine** | HMDB11738 | -3.20/-4.90 |  |  |
| M259T51 [M-H]^-^  Monoisotopic mass :  260.119536  Calcd. mass : 260.112070 | **Asparaginyl-Glutamine** | HMDB28729 | -3.50/-6.00 | 3.0 | 6.1E-14 |
|  | **Asparaginyl-Gamma-glutamate** | HMDB28745 | -3.40/-5.80 |  |  |
|  | **Glutaminyl-Asparagine** | HMDB28792 | -3.50/-6.00 |  |  |
|  | **Gamma-glutamyl-Asparagine** | HMDB29144 | -3.50/-6.00 |  |  |
| M259T51 [M-H_2_O-H]^-^  Monoisotopic mass :  278.134816  Calcd. mass : 278.130028 | **Pantetheine** | HMDB03426 | -0.10/-1.50 |  |  |
| M259T51  [M-H_2_O+HCOOH-H]^-^  Monoisotopic mass :  232.109436  Calcd. mass : 232.105922 | **N_2_-Succinyl-L-ornithine** | HMDB01199 | -3.40/-4.10 |  |  |
|  | **4-(Glutamylamino)butanoate** | HMDB12161 | -3.40/-3.90 |  |  |
|  | **Aspartyl-Valine** | HMDB28766 | -3.00/-3.50 |  |  |
|  | **Hydroxyprolyl-Threonine** | HMDB28873 | -3.30/-4.90 |  |  |
|  | **Threoninyl-Hydroxyproline** | HMDB29062 | -3.10/-4.90 |  |  |
|  | **Valyl-Aspartate** | HMDB29123 | -3.00/-3.20 |  |  |
| M262T468 [M-H_2_O-H]^-^  Monoisotopic mass :  281.092996  Calcd. mass : 281.089937 | **4-hydroxyphenyl**  **acetylglutamine** | HMDB06061 | 0.72/0.42 | 2.0 | 2.9E-8 |
| M262T468  [M-H_2_O+HCOOH-H]^-^  Monoisotopic mass :  235.067616  Calcd. mass : 235.062677 | **Asparaginyl-Cysteine** | HMDB28728 | -3.00/-4.80 |  |  |
|  | **Cysteinyl-Asparagine** | HMDB28770 | -2.90/-4.80 |  |  |
| M267T84 [M-H]^-^  Monoisotopic mass :  268.099015  Calcd. mass : 268.105922 | **Serinyl-Tyrosine** | HMDB28728 | -3.00/-4.80 | 2.6 | 4.0E-13 |
|  | **Tyrosyl-Serine** | HMDB29114 | -2.40/-3.10 |  |  |
| M267T84 [M-H_2_O-H]^-^  Monoisotopic mass :  286.114295  Calcd. mass : 286.109961 | **Histidinyl-Methionine** | HMDB28891 | -2.20/-3.30 |  |  |
|  | **Methionyl-Histidine** | HMDB28975 | -2.20/-3.40 |  |  |
| M272T48 [M-H]^-^  Monoisotopic mass :  273.127608  Calcd. mass : 273.121237 | **Glutaconylcarnitine** | HMDB13129 | -1.70/-3.90 | 3.8 | 2.7E-12 |
| M272T48  [M-H_2_O+HCOOH-H]^-^  Monoisotopic mass :  245.117508  Calcd. mass : 245.113747 | **Glutamyl-Valine** | HMDB28832 | -2.50/-3.10 |  |  |
|  | **Valyl-Glutamate** | HMDB29126 | -2.60/-3.10 |  |  |
| M275T336 [M-H]^-^  Monoisotopic mass :  276.111985  Calcd. mass : 276.106984 | **5-amino-6-ribitylamino uracil** | HMDB11106 | -2.40/-4.40 | 4.4 | 0 |
| M275T336 [M-H]^-^  Monoisotopic mass :  276.111985  Calcd. mass : 276.111007 | **N-lactoyl-Tryptophan** | HMDB62178 | 0.85/0.75 |  |  |
| M275T336 [M-H_2_O-H]^-^  Monoisotopic mass :  294.127265  Calcd. mass : 294.121572 | **Glutamylphenylalanine** | HMDB00594 | -2.10/-2.10 |  |  |
|  | **Hydroxyprolyl-Tyrosine** | HMDB28875 | -2.00/-2.90 |  |  |
|  | **Tyrosyl-Hydroxyproline** | HMDB29106 | -2.20/-2.90 |  |  |
| M277T54  Monoisotopic mass : 277.017763 | No hit | -- | -- | 3.0 | 9.3E-15 |
| M281T214  Monoisotopic mass :  281.097271 | No hit | -- | -- | 8.3 | 1.3E-7 |
| M288T244  [M-H_2_O+HCOOH-H]^-^  Monoisotopic mass :  261.107082  Calcd mass : 261.111341 | Glycyl-Tryptophan | HMDB28852 | -1.20/-2.20 | 2.0 | 1.5E-7 |
|  | Tryptophyl-Glycine | HMDB29083 | -0.94/-2.20 |  |  |
| M289T101/M289T126  /M289T377  [M-H_2_O+HCOOH-H]^-^  Monoisotopic mass :  262.099769/  262.099765/  262.099796  Calcd mass : 262.098728 | Hydroxyprolyl-Methionine | HMDB28869 | -1.90/-3.60 | 2.8/  3.2/  2.8 | 8.2E-14/  1.3E-11/  1.3E-12 |
|  | Methionyl-Hydroxyproline | HMDB28974 | -2.00/-3.60 |  |  |
| M290T101  [M-H]^-^  Monoisotopic mass :  291.113302  Calcd mass : 291.121906 | Serinyl-Tryptophan | HMDB29050 | -1.50/-2.70 | 2.8 | 1.7E-13 |
|  | Tryptophyl-Serine | HMDB29092 | -1.50/-2.70 |  |  |
| M290T101  [M-H_2_O-H]^-^  Monoisotopic mass :  309.128582  Calcd mass : 309.132471 | Gamma-Glutamyltyrosine | HMDB11741 | -2.30/-2.50 |  |  |
| M297T462  Monoisotopic mass :  297.138817 | No hit | -- | -- | 2.0 | 8.9E-8 |
| M299T419  Monoisotopic mass :  299.070613 | No hit | -- | -- | 2.3 | 7.8E-10 |
| M305T953  [M-H]^-^  Monoisotopic mass :  306.031328  Calcd mass : 306.025302 | Uridine 2’,3’-cyclic phosphate | HMDB11640 | -1.30/-1.60 | 2.1 | 1.0E-12 |
| M310T412  Monoisotopic mass :  310.151118 | No hit | -- | -- | 3.2 | 2.2E-15 |
| M333T101  Monoisotopic mass :  333.091904 | No hit | -- | -- | 2.9 | 1.5E-14 |
| M334T126  [M-H_2_O+HCOOH-H]^-^  Monoisotopic mass :  307.093063  Calcd mass : 307.0911995 | (S)-Succinyldihydrolipoamide | HMDB01177 | 1.97/1.06 | 3.2 | 2.1E-12 |
| M334T126  [M-H_2_O+HCOOH-H]^-^  Monoisotopic mass :  307.093063  Calcd mass : 307.099062 | 3’-UMP | HMDB60282 | -1.80/-2.20 |  |  |
| M351T63  Monoisotopic mass :  351.033709 | No hit | -- | -- | 3.0 | 7.0E-12 |
| M353T92  Monoisotopic mass :  353.049311 | No hit | -- | -- | 2.6 | 1.2E-12 |
| M356T92  [M-H_2_O+HCOOH-H]^-^  Monoisotopic mass :  329.045316  Calcd mass : 329.052520 | Cyclic AMP | HMDB00058 | -2.30/-3.80 | 2.5 | 4.3E-12 |
| M356T421  Monoisotopic mass :  356.159608 | No hit | -- | -- | 2.5 | 1.5E-11 |
| M370T376  Monoisotopic mass :  370.071777 | No hit | -- | -- | 3.1 | 3.3E-14 |
| M371T378  [M-H_2_O+HCOOH-H]^-^  Monoisotopic mass :  344.063122  Calcd mass : 344.070812 | Thiamine monophosphate | HMDB02666 | -2.30/-3.80 | 2.9 | 1.8E-13 |
| M383T468  Monoisotopic mass :  383.123116 | No hit | -- | -- | 3.6 | 0 |
| M387T953  Monoisotopic mass :  387.026688 | No hit | -- | -- | 2.5 | 5.2E-14 |
| M403T967  Monoisotopic mass :  403.000590 | No hit | -- | -- | 3.3 | 1.3E-15 |
| M412T306  [M-H_2_O+HCOOH-H]^-^  Monoisotopic mass :  385.108981  Calcd mass : 385.113783 | Phosphatidylserine | HMDB14291 | -1.00/-1.60 | 3.7 | 2.2E-16 |
| M452T373  Monoisotopic mass :  452.096238 | No hit | -- | -- | 2.8 | 5.6E-14 |
| M453T375  [M-H_2_O+HCOOH-H]^-^  Monoisotopic mass :  426.097125  Calcd mass : 426.087905 | Cysteineglutathione disulfide | HMDB00656 | -2.90/-8.00 | 6.9 | 0 |
| M459T304  Monoisotopic mass :  459.146440 | No hit | -- | -- | 5.0 | 4.4E-8 |
| M469T953  Monoisotopic mass :  469.029665 | No hit | -- | -- | 2.9 | 2.2E-15 |
| M483T59  Monoisotopic mass :  483.179233 | No hit | -- | -- | 3.3 | 8.0E-13 |
| M485T966  Monoisotopic mass :  485.003743 | No hit | -- | -- | 3.8 | 0 |
| M513T347  Monoisotopic mass :  513.155399 | No hit | -- | -- | 3.4 | 1.3E-15 |
| M526T333  Monoisotopic mass :  526.165866 | No hit | -- | -- | 2.3 | 1.6E-10 |
| M551T953  Monoisotopic mass :  551.033080 | No hit | -- | -- | 3.1 | 4.4E-16 |
| M567T965  Monoisotopic mass :  567.007168 | No hit | -- | -- | 4.9 | 0 |
| M633T953  Monoisotopic mass :  633.036596 | No hit | -- | -- | 3.6 | 0 |
| M649T964  Monoisotopic mass :  649.010587 | No hit | -- | -- | 6.0 | 0 |
| M667T315  Monoisotopic mass :  667.191794 | No hit | -- | -- | 3.0 | 1.3E-13 |
| M687T99  Monoisotopic mass :  687.148750 | No hit | -- | -- | 52.5 | 2.0E-12 |
| M709T101  Monoisotopic mass :  709.130638 | No hit | -- | -- | 4.9 | 5.1E-14 |
| M715T953  Monoisotopic mass :  715.040112 | No hit | -- | -- | 4.0 | 0 |
| M731T963  Monoisotopic mass :  731.013678 | No hit | -- | -- | 14.1 | 0 |
| M797T953  Monoisotopic mass :  797.043411 | No hit | -- | -- | 5.1 | 0 |
| M879T954  [M-H_2_O+HCOOH-H]^-^  Monoisotopic mass :  852.044011  Calcd mass : 852.043179 | Guanosine tetraphosphate adenosine | HMDB01454 | -0.40/-8.40 | 7.1 | 2.2E-16 |


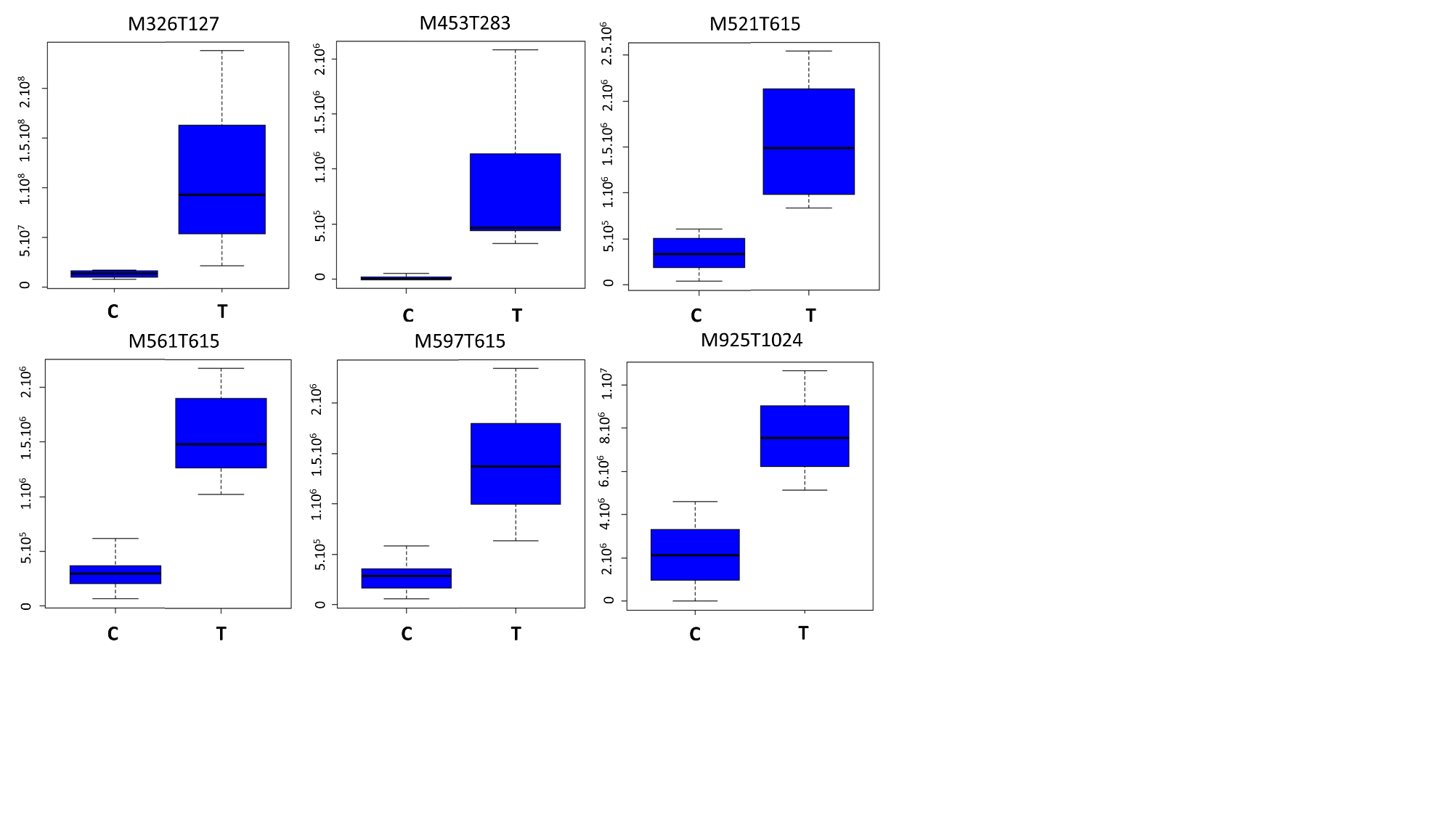


**Fig. S1** Box and Whiskers plots of the candidate biomarkers of exposure to millimeter waves appearing in positive-ion mode endocellular lipidomics.


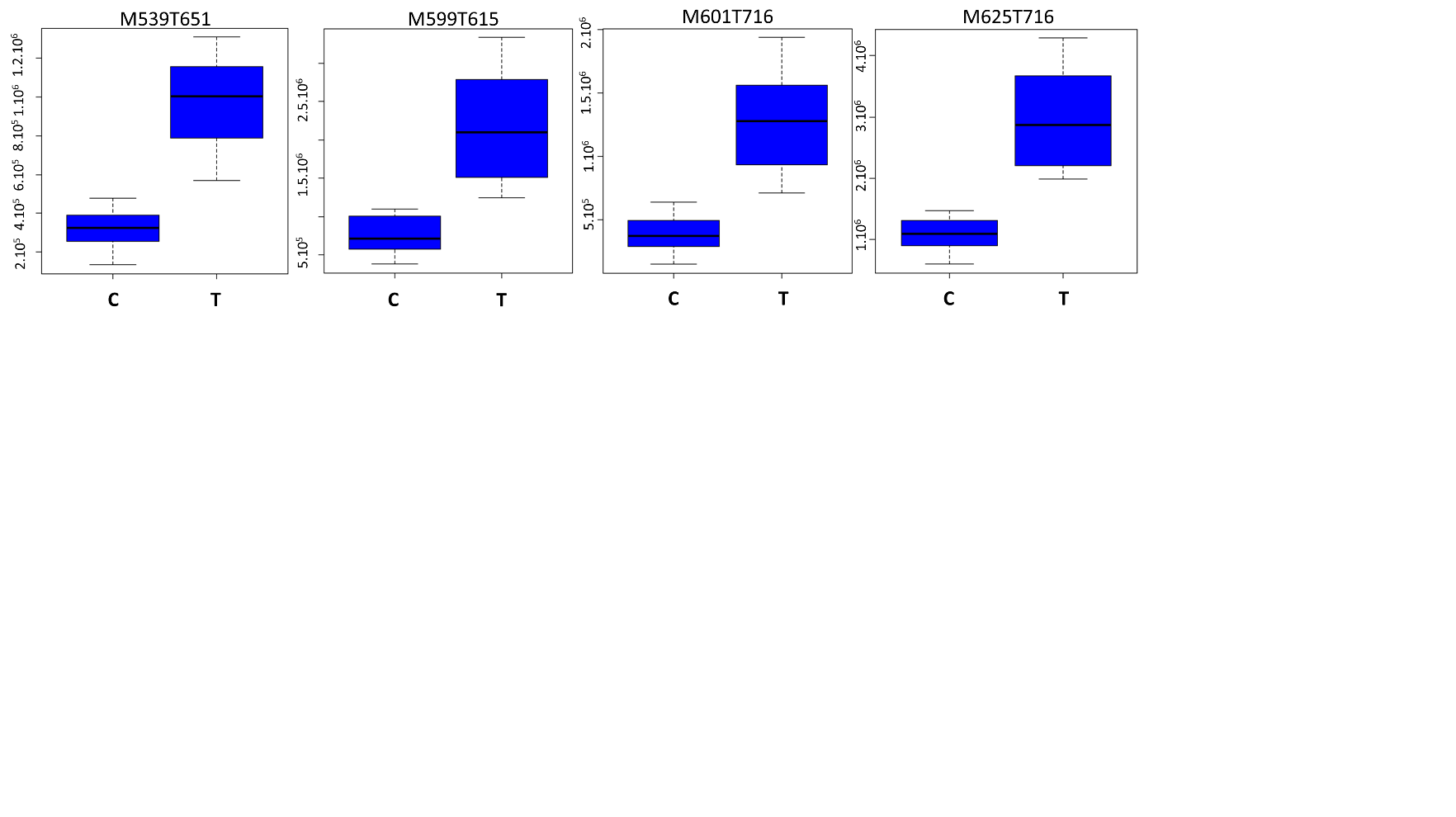


**Fig. S2** Box and Whiskers plots of the dysregulated features upon millimeter waves exposure as appearing in negative polarity endocellular lipidomics.


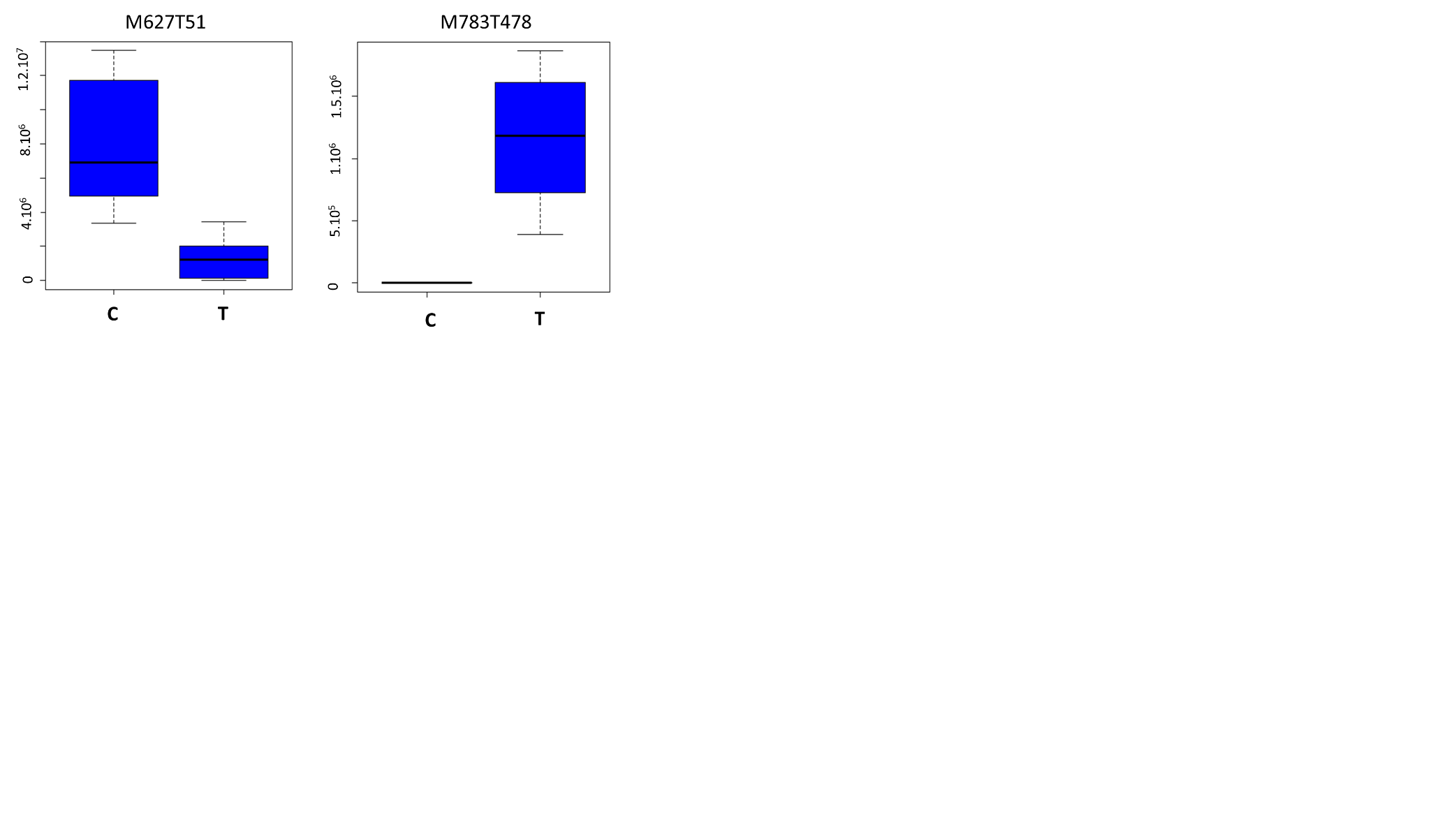


**Fig. S3** Box and Whiskers plots of the putative biomarkers of exposure to millimeter waves observed throughout positive-ion mode exocellular lipidomics.


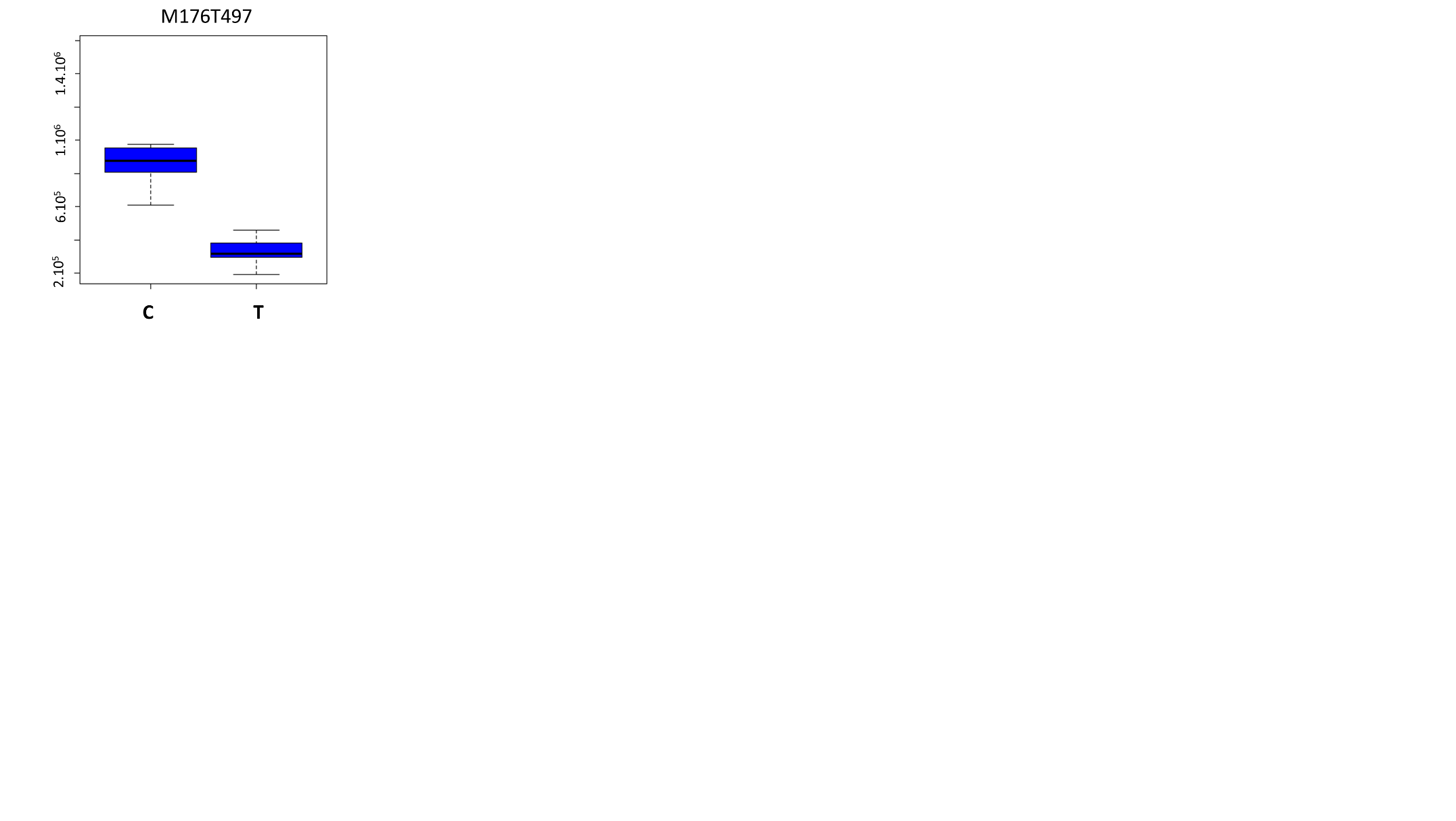


**Fig. S4** Box and Whiskers Plot of M176T497, the only frame which appeared to be dysregulated following millimeter waves exposure in positive-ion mode endocellular metabolomics.


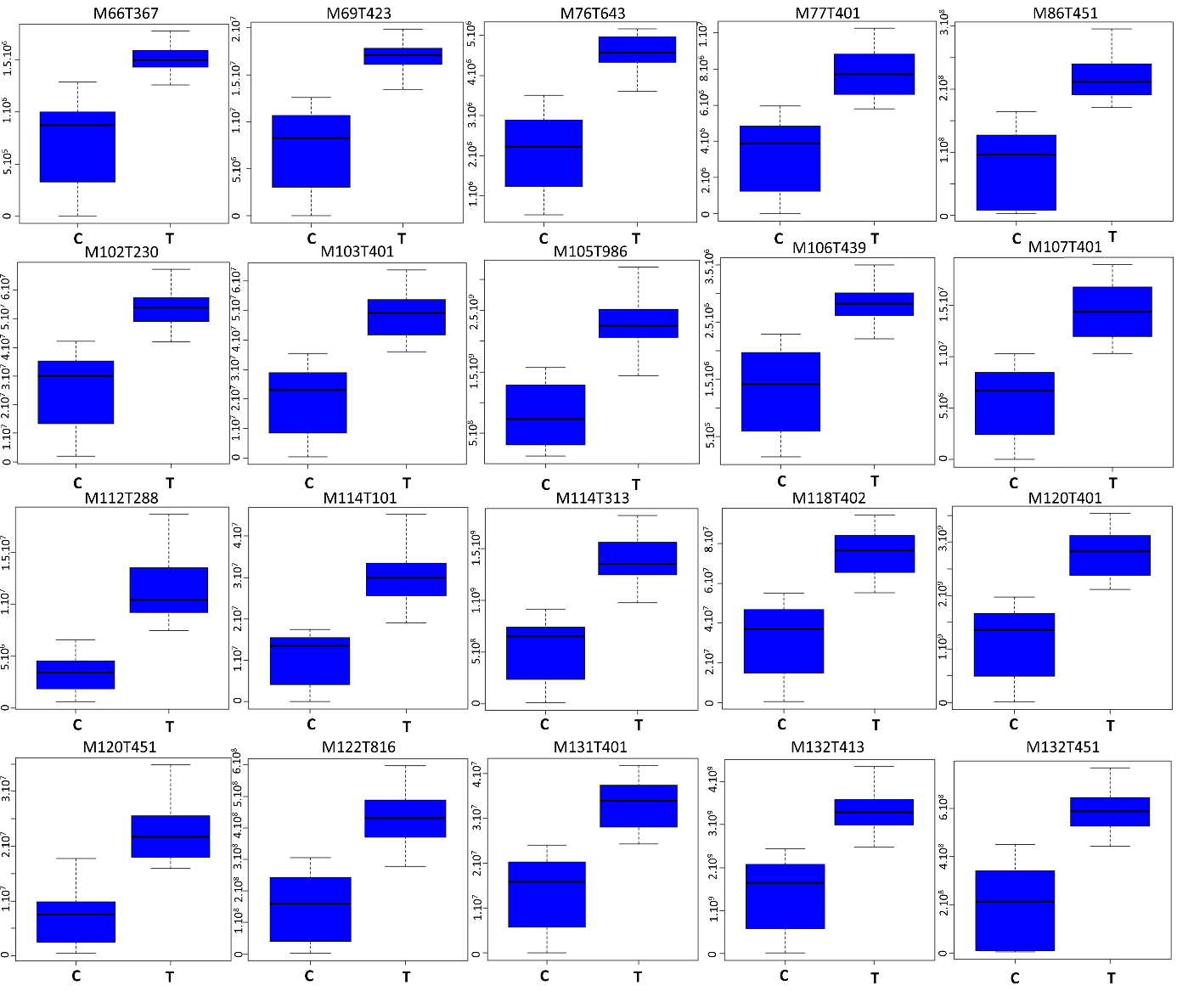


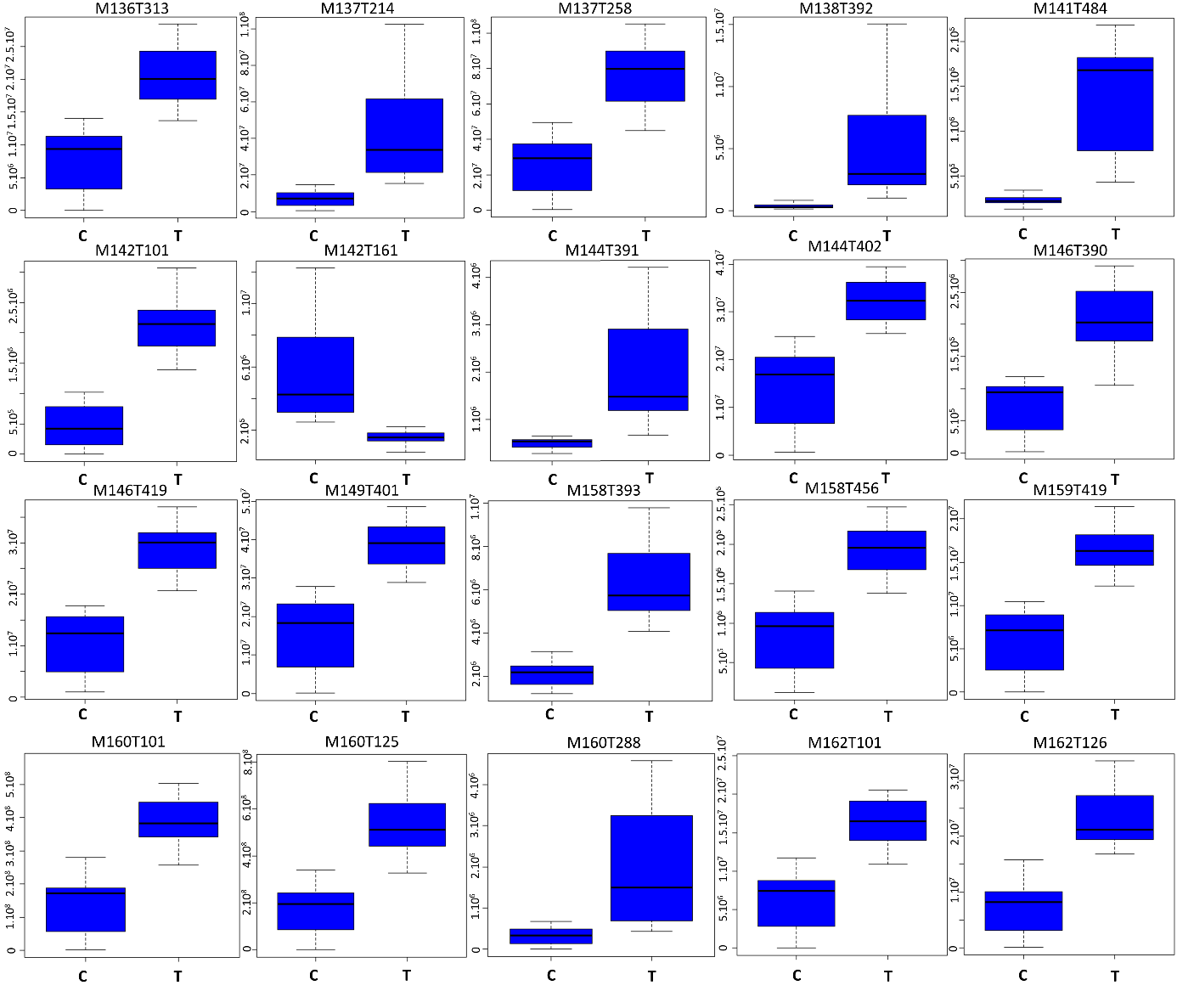


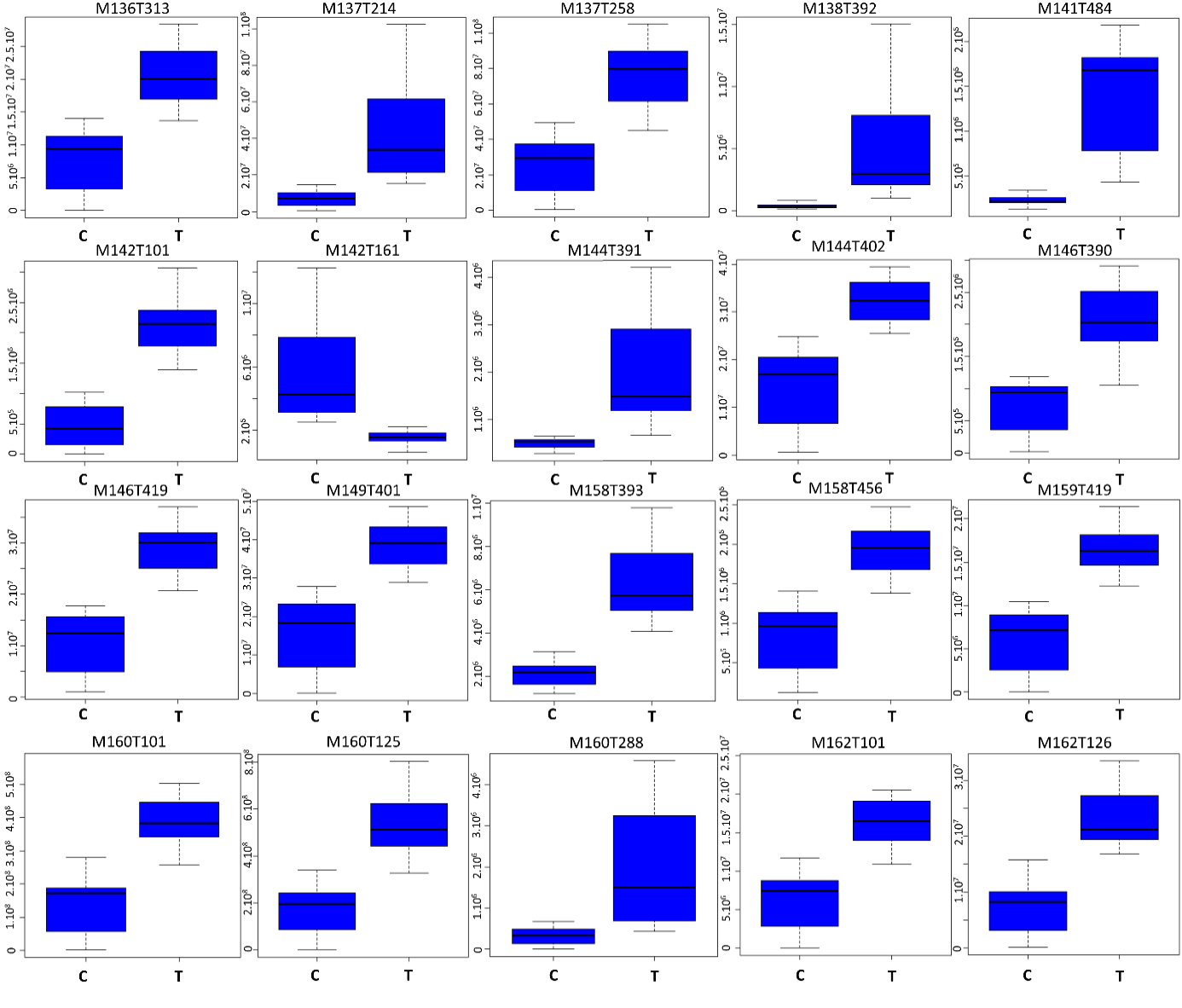


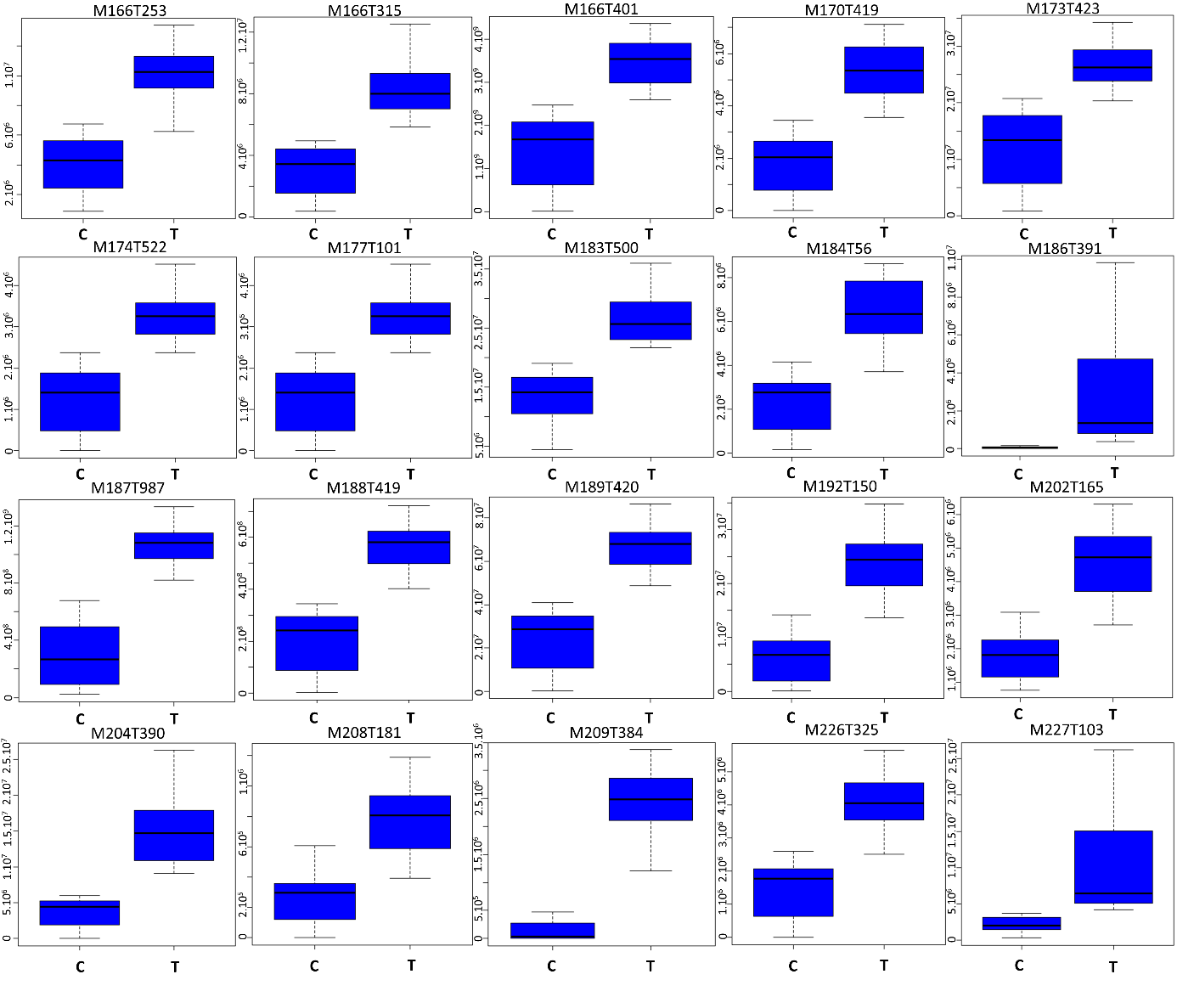


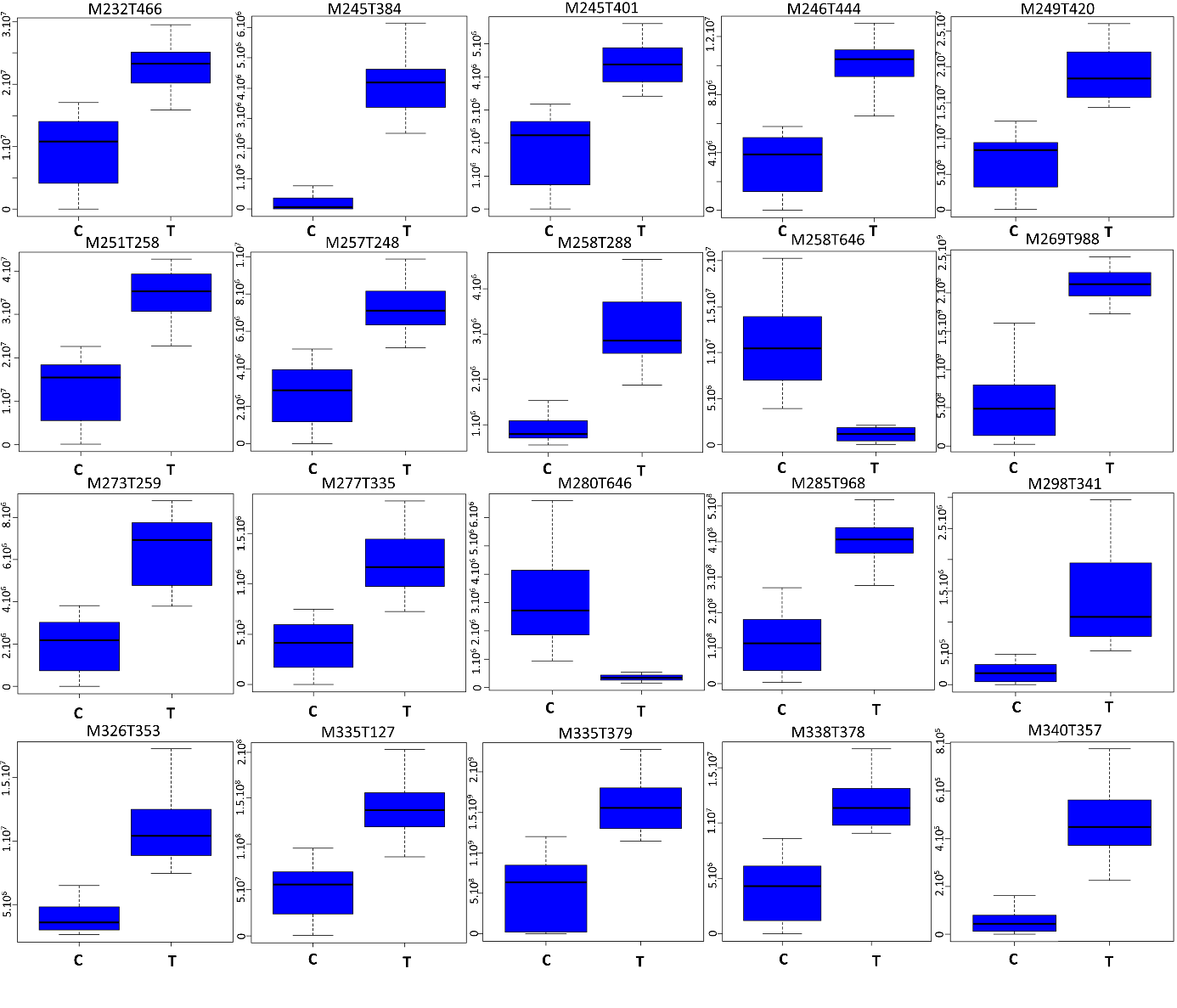


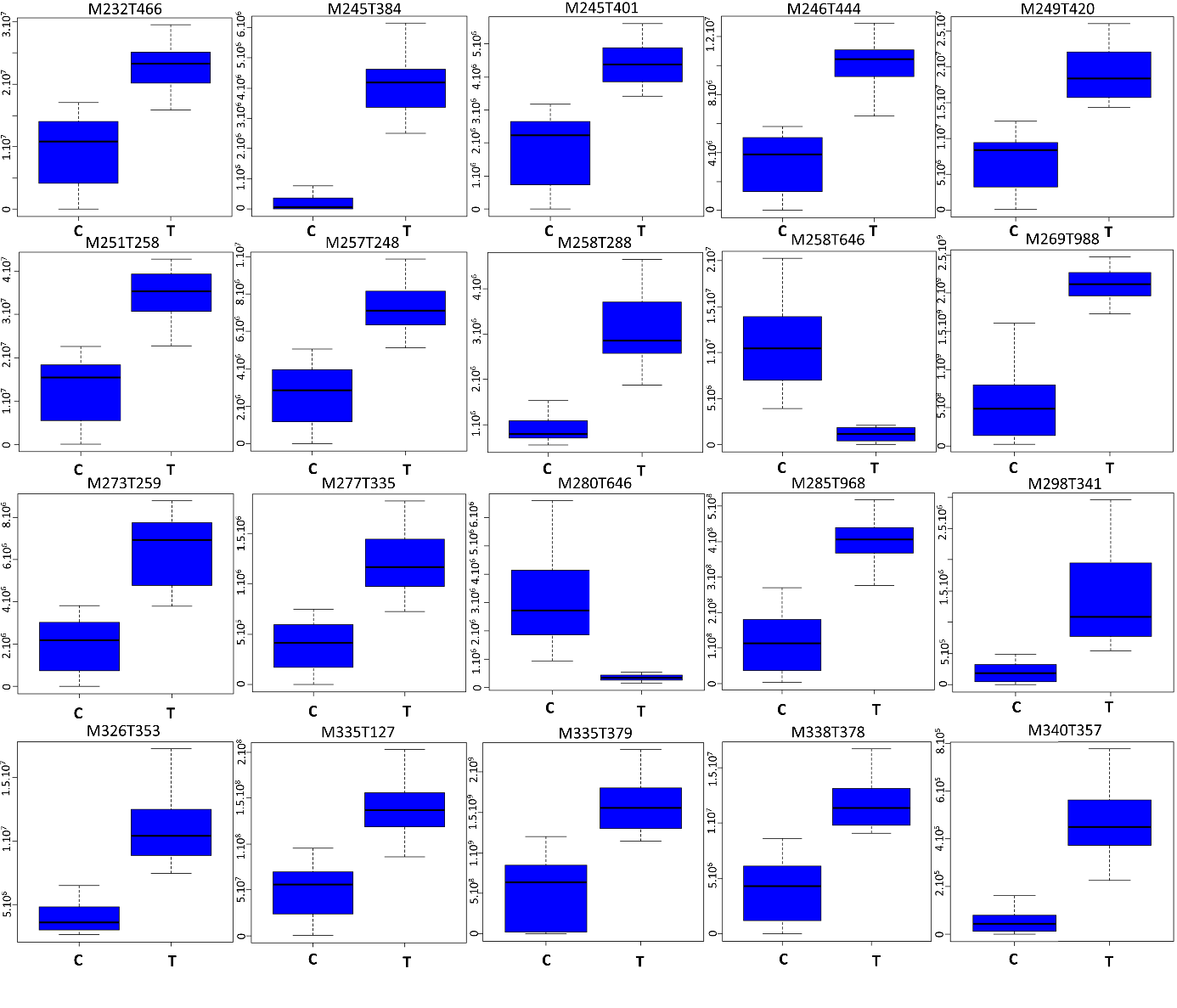

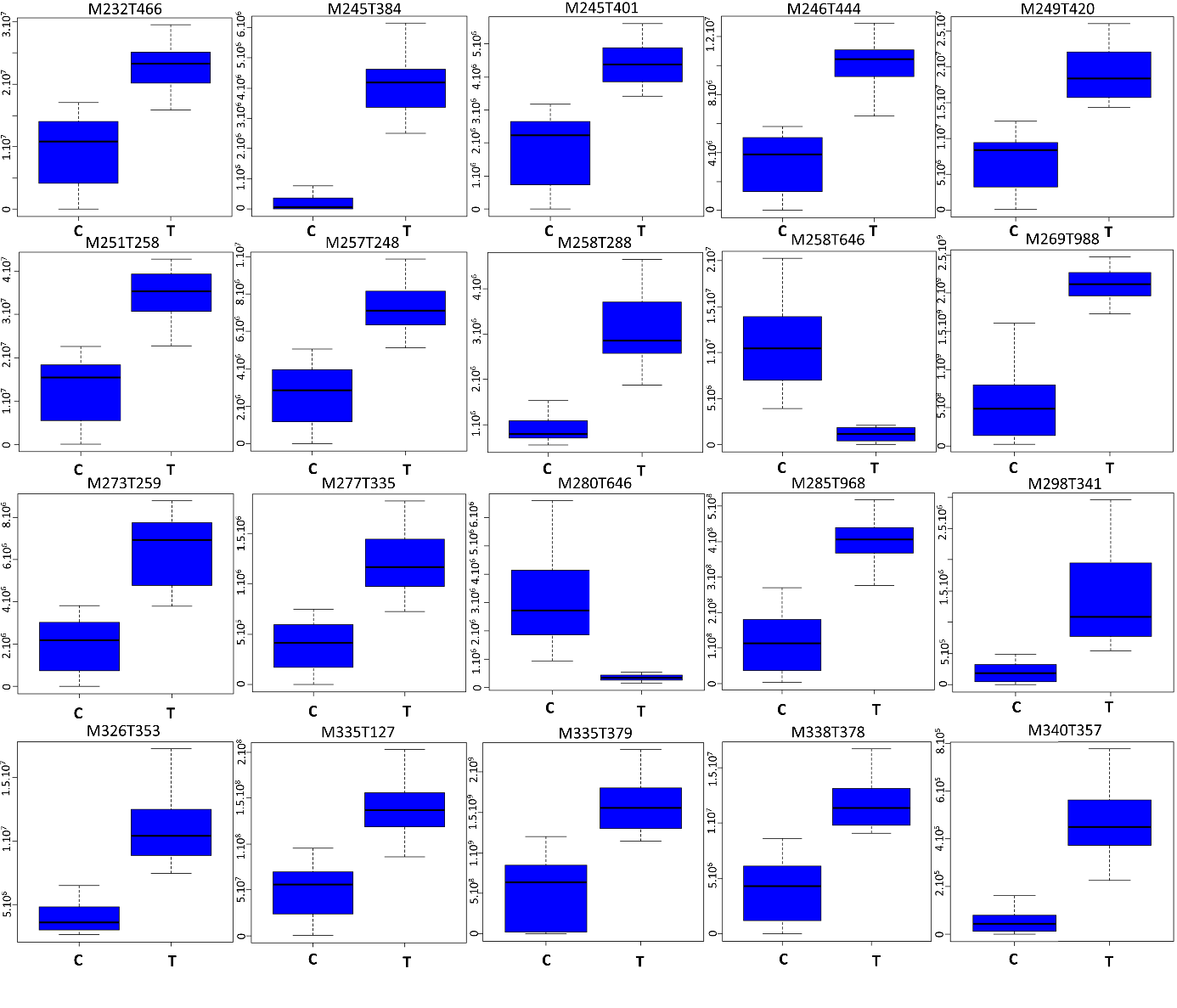

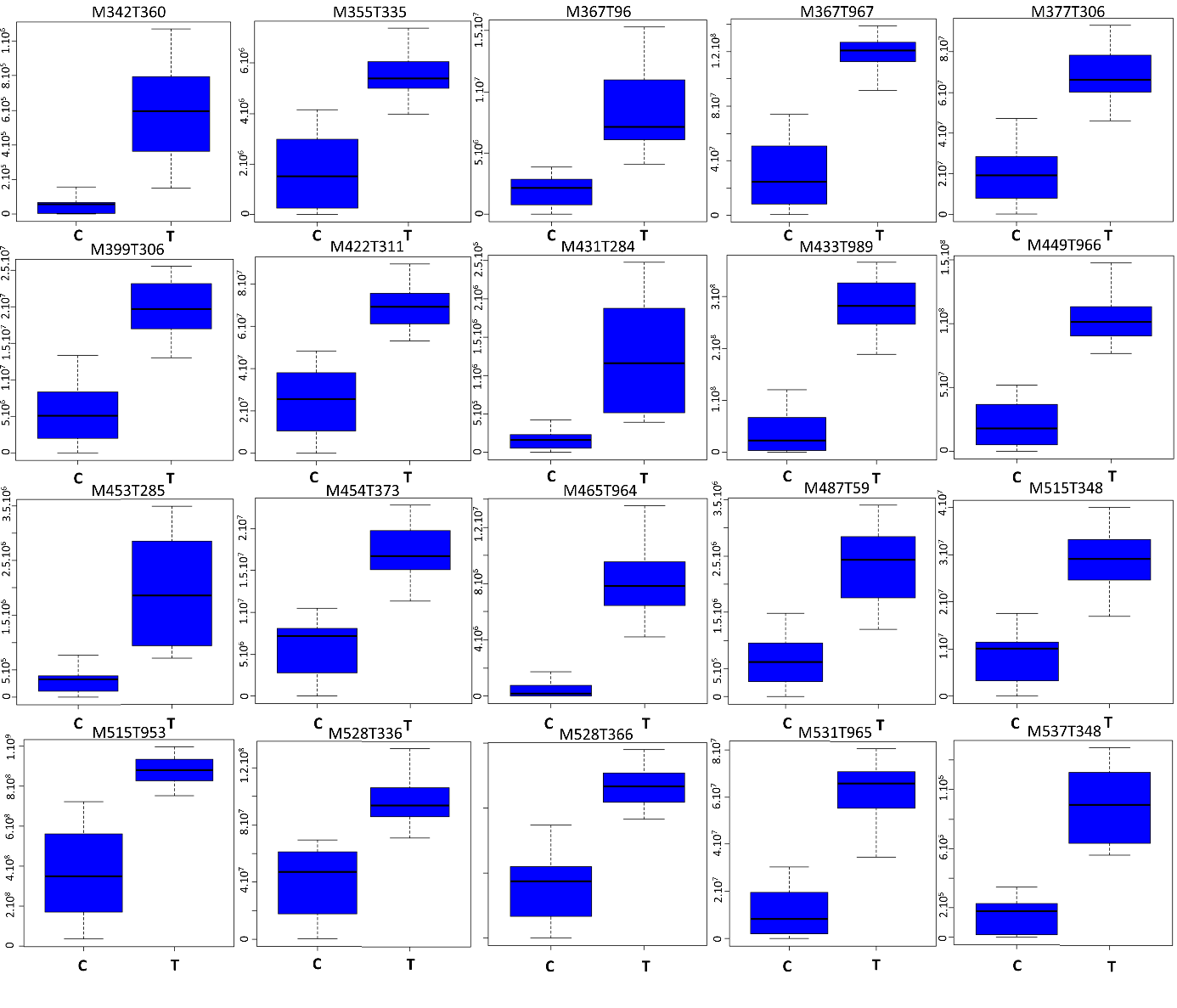


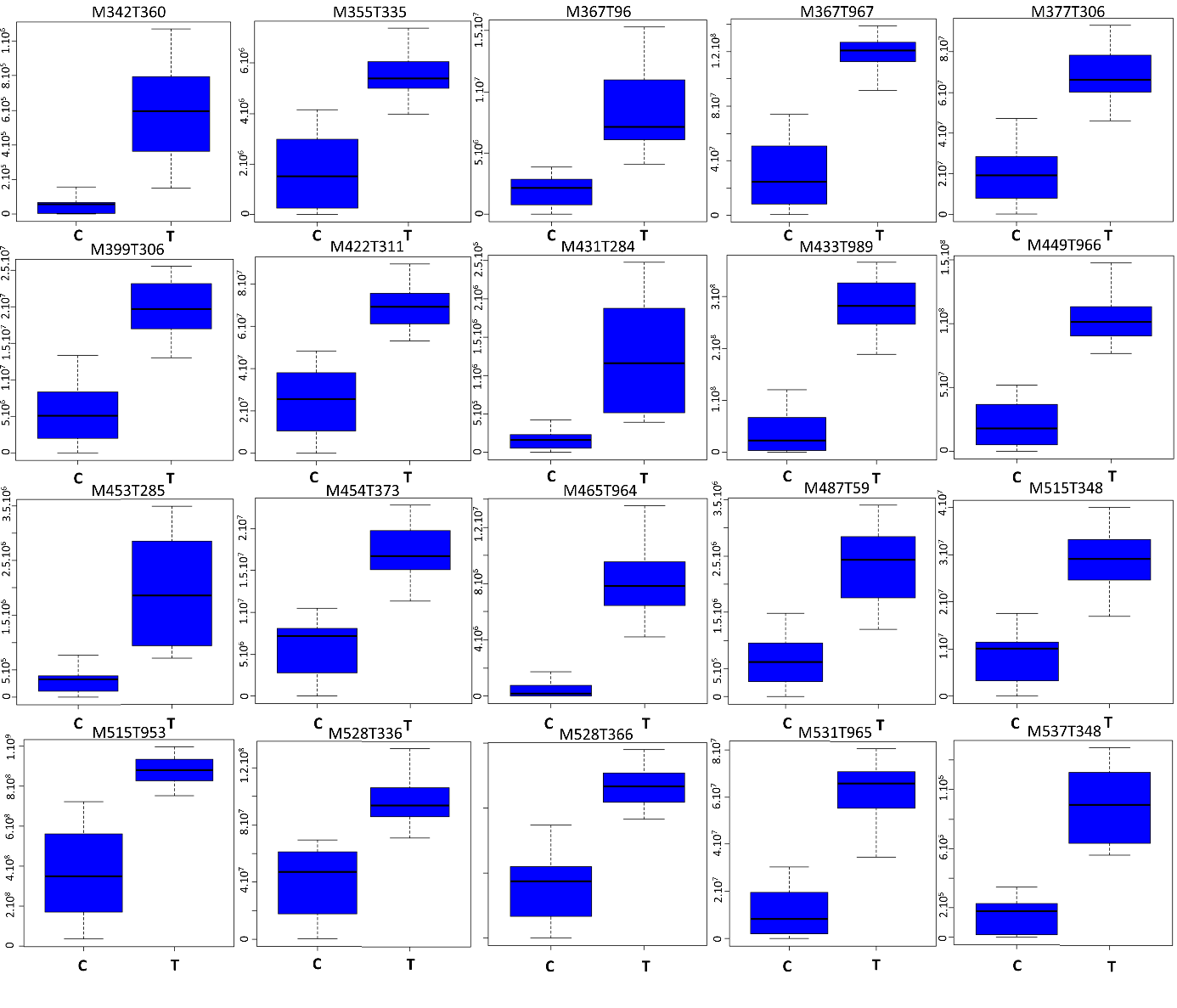

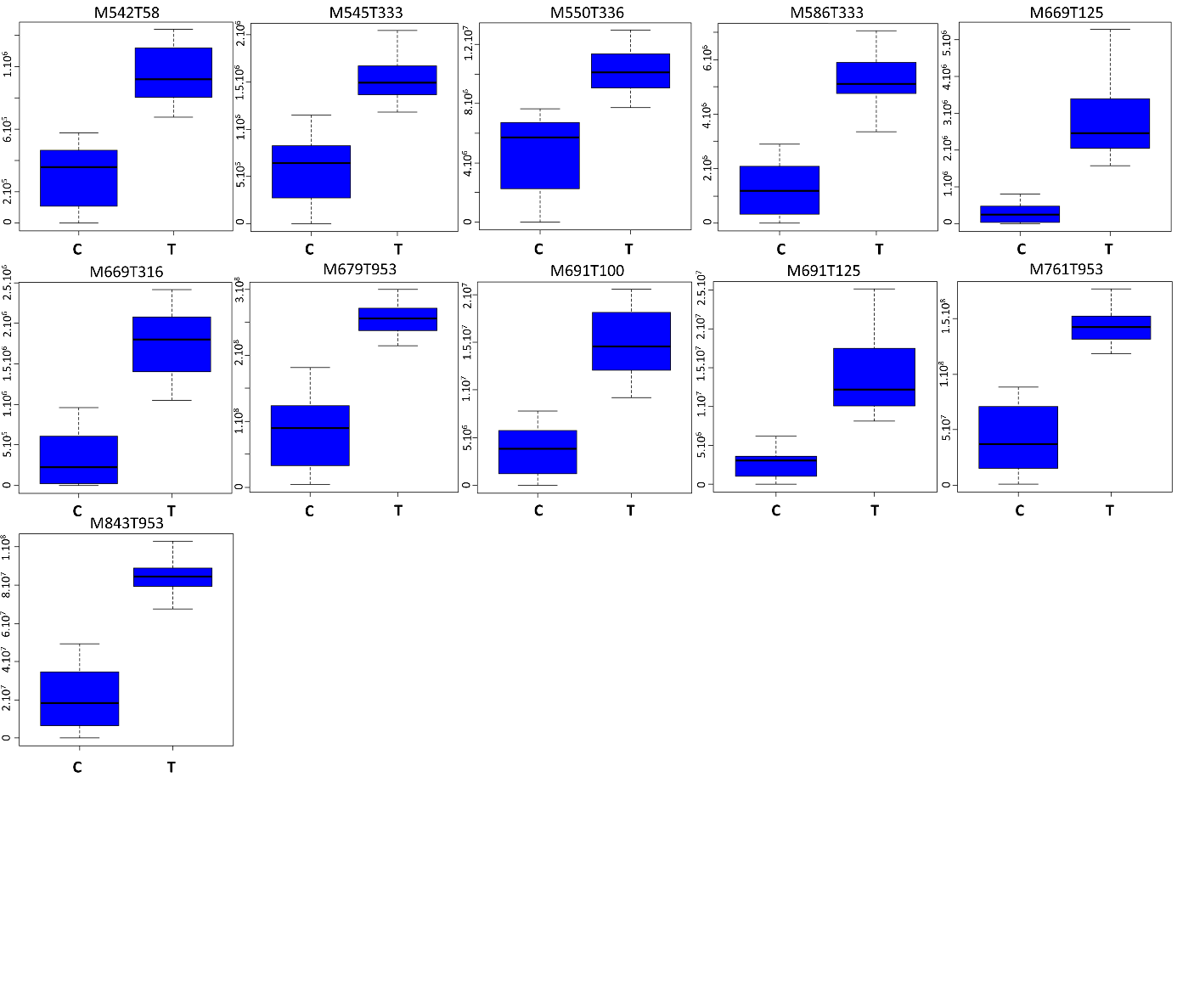


**Fig. S5** Box and Whiskers Plots of the dysregulated features upon exposure to millimeter waves as revealed throughout positive-ion mode exocellular metabolomics.


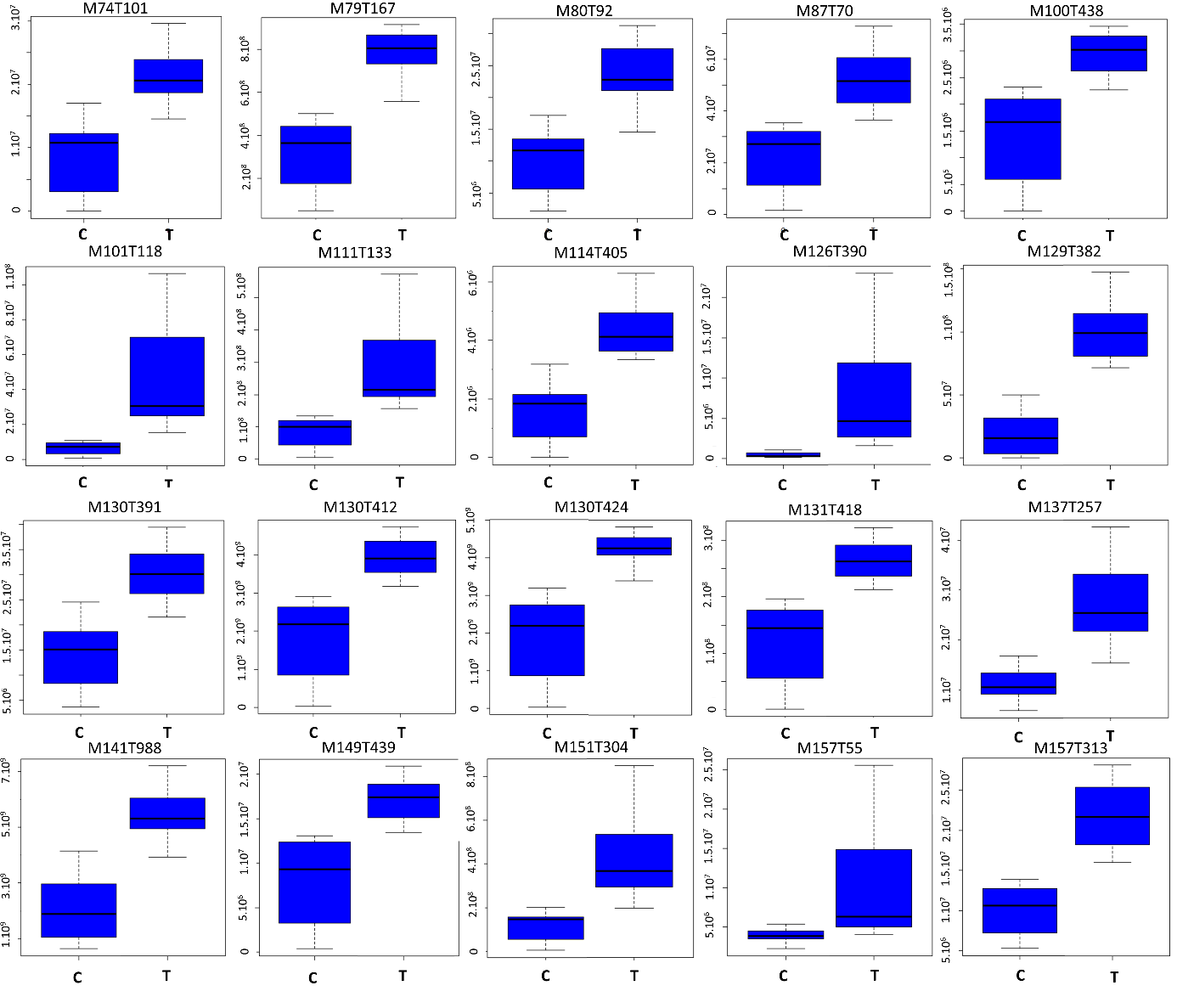

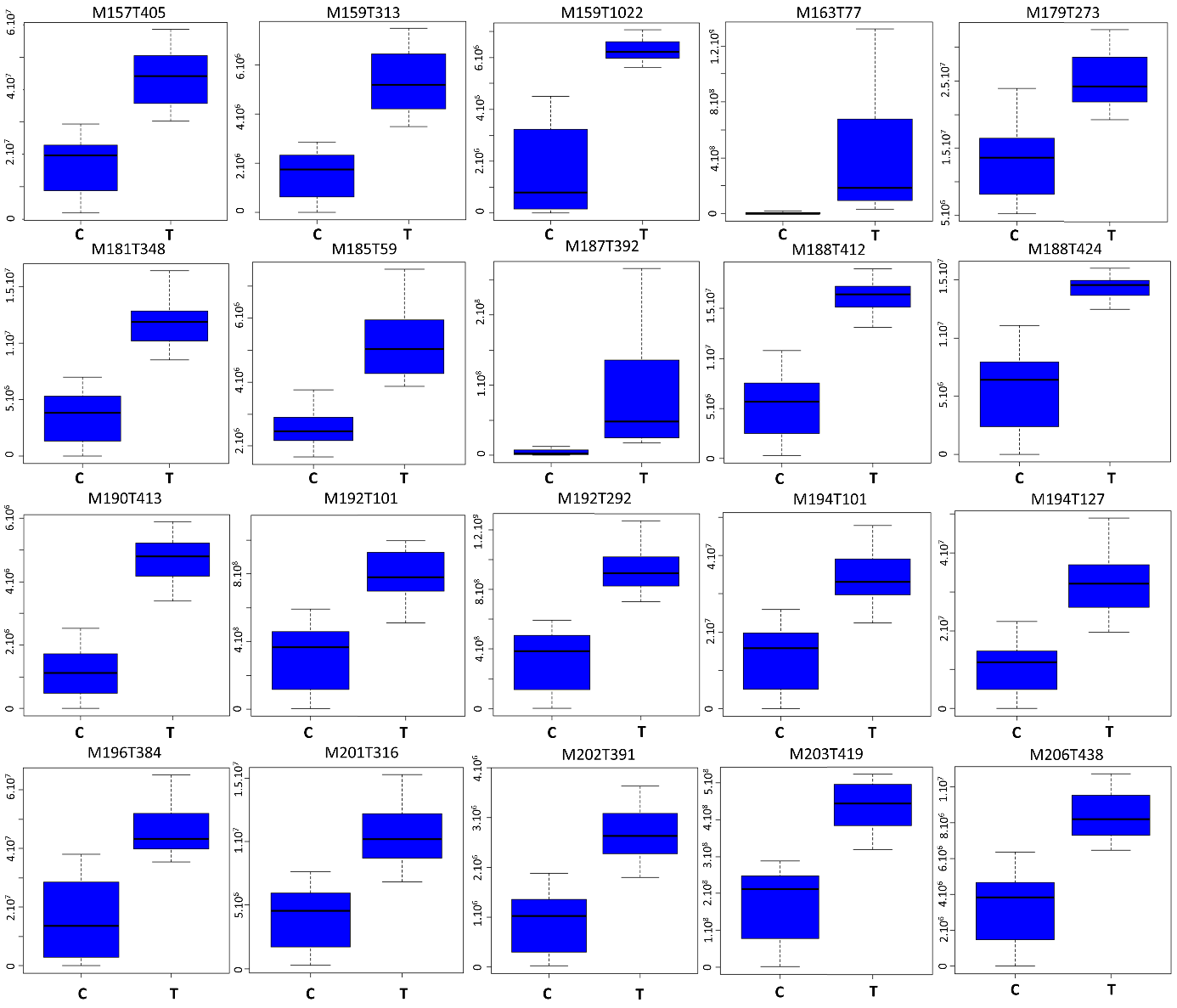


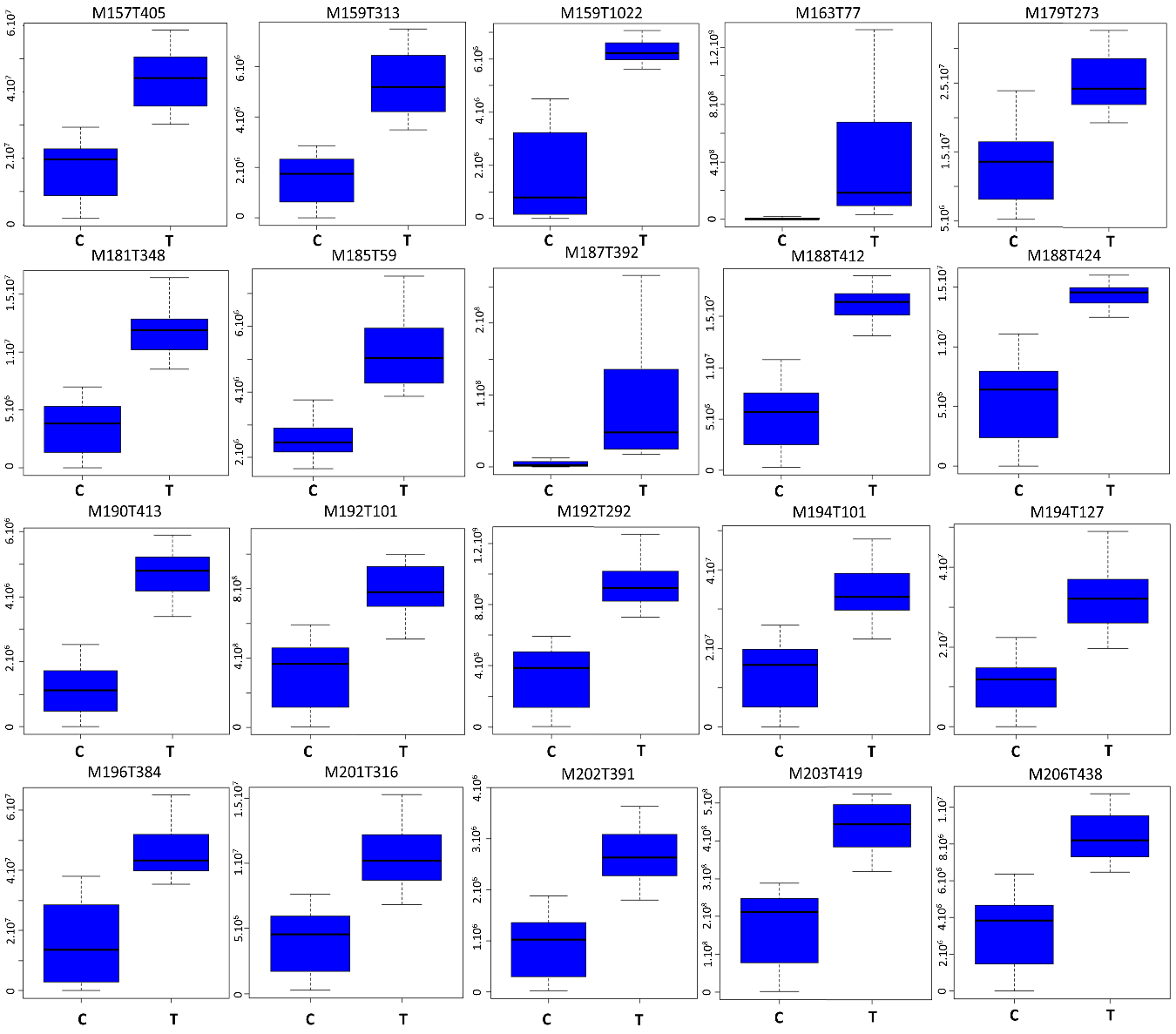

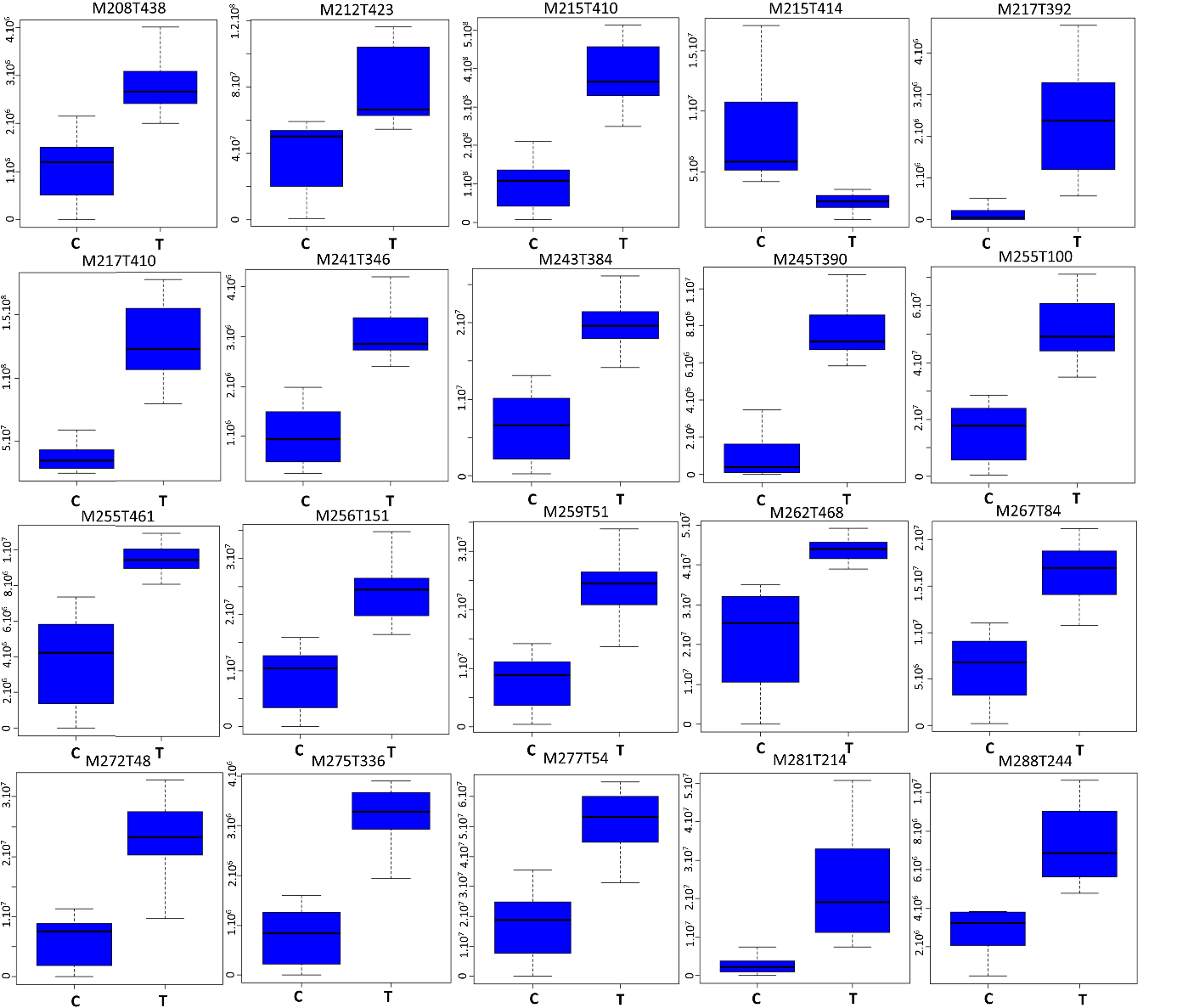

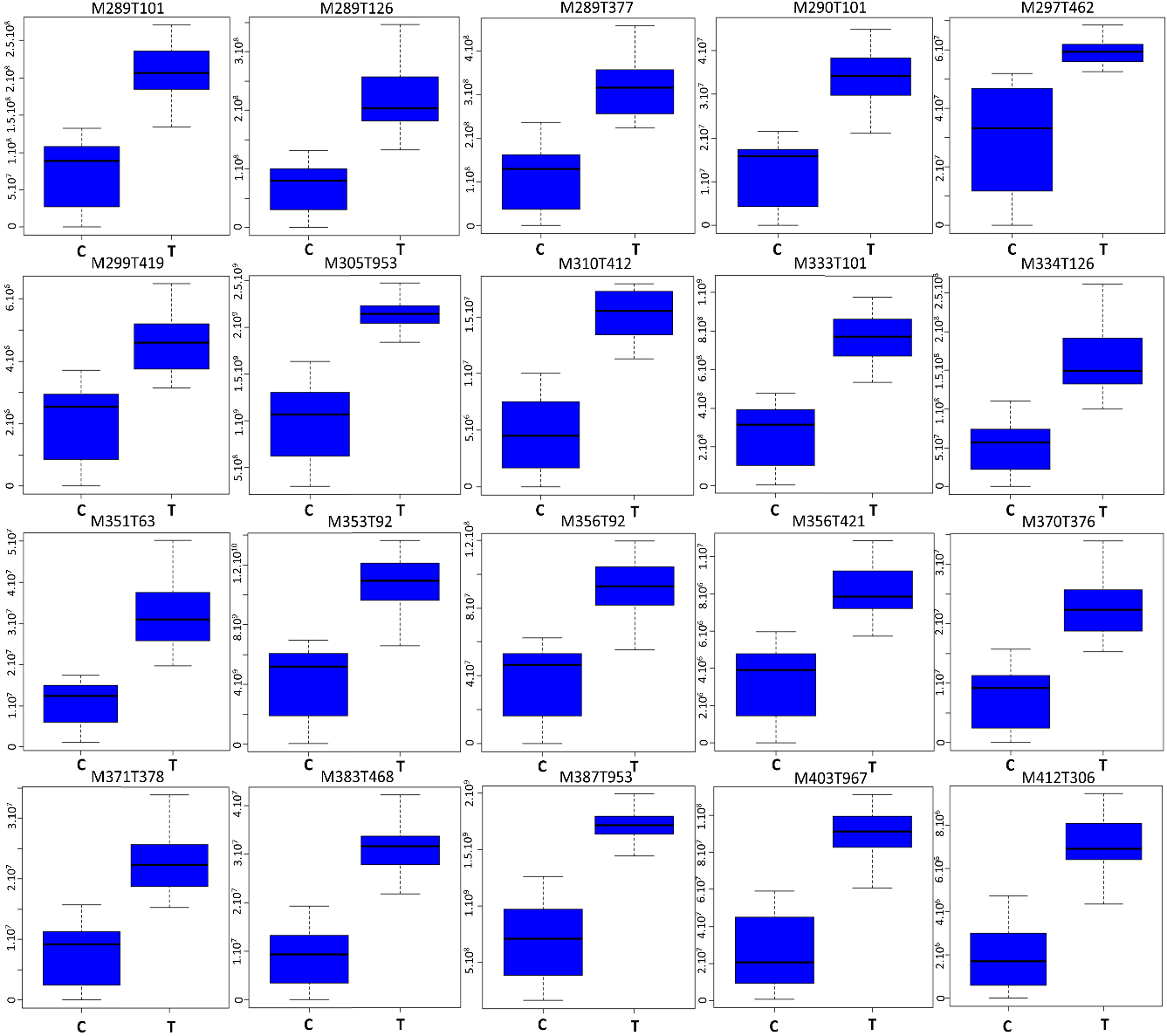

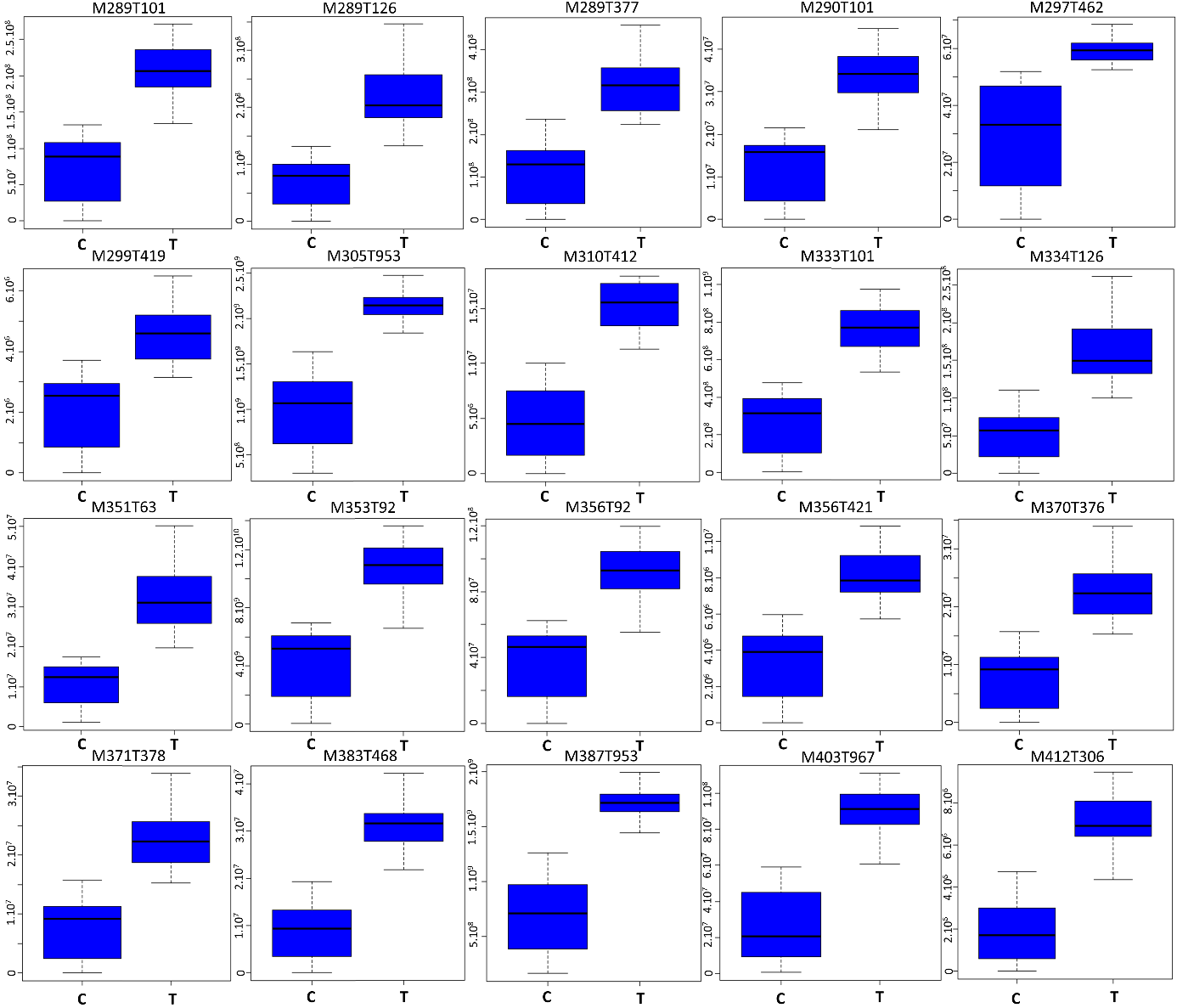

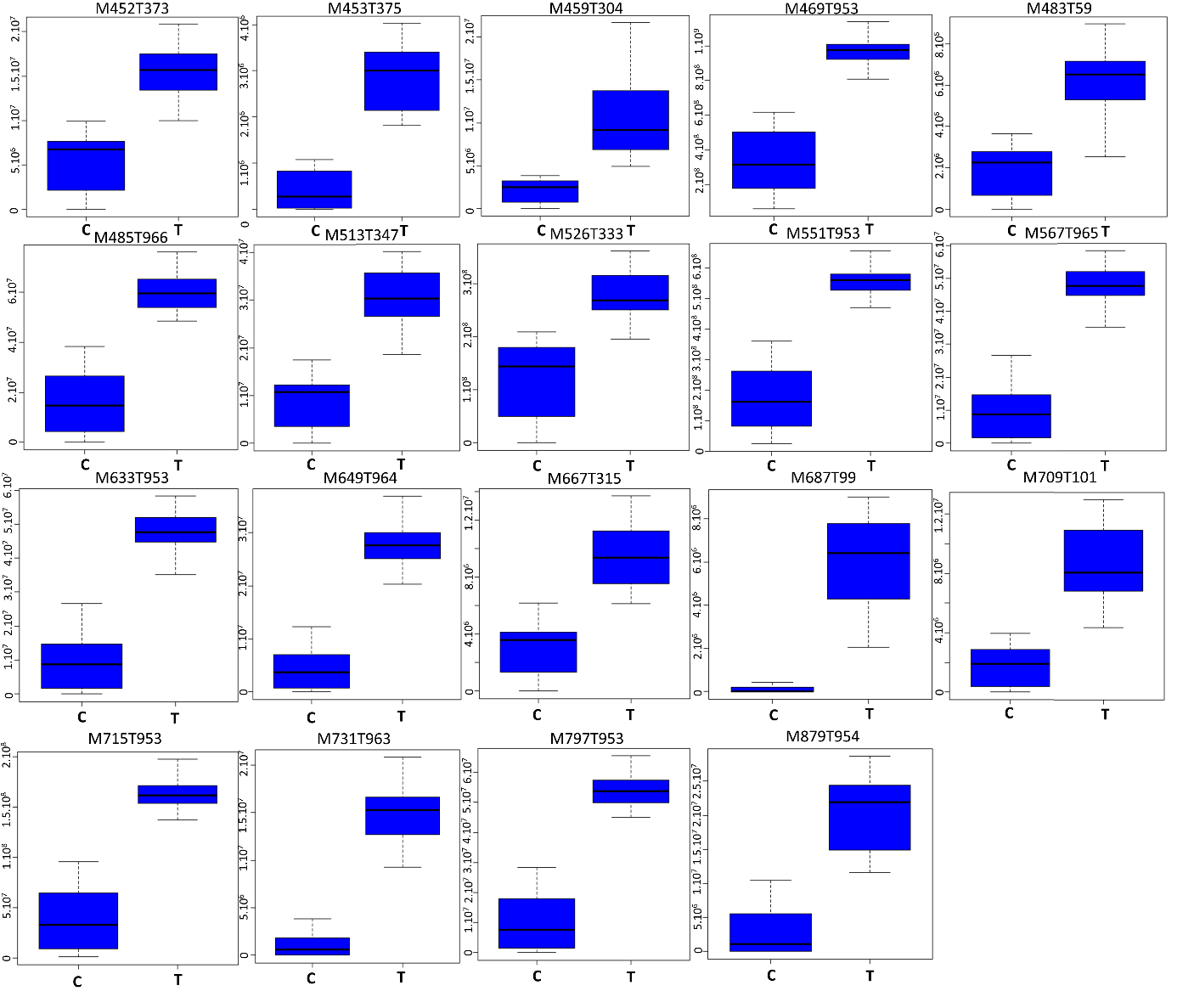


**Fig. S6** Box and Whiskers Plots of the dysregulated features upon exposure to millimeter waves as revealed throughout negative-ion mode exocellular metabolomics.
